# Assessing the therapeutic potential of *Graptopetalum paraguayense* on Alzheimer’s disease using patient iPSC-derived neurons

**DOI:** 10.1038/s41598-019-55614-9

**Published:** 2019-12-17

**Authors:** Pei-Chun Wu, Ming-Ji Fann, Tu Thanh Tran, Shu-Cian Chen, Tania Devina, Irene Han-Juo Cheng, Cheng-Chang Lien, Lung-Sen Kao, Shuu-Jiun Wang, Jong-Ling Fuh, Tsai-Teng Tzeng, Chi-Ying Huang, Young-Ji Shiao, Yu-Hui Wong

**Affiliations:** 1 Brain Research Center, National Yang-Ming University, Taipei, 11221 Taiwan (ROC); 2 Department of Life Sciences and Institute of Genome Sciences, National Yang-Ming University, Taipei, 11221 Taiwan (ROC); 3 Division of General Neurology, Neurological Institute, Taipei Veterans Hospital, Taipei, 11217 Taiwan (ROC); 4 Institute of Biopharmaceutical Sciences, National Yang-Ming University, Taipei, 11221 Taiwan (ROC); 5 National Research Institute of Chinese Medicine, Ministry of Health and Welfare, Taipei, 11221 Taiwan (ROC); 6 Institute of Brain Science, National Yang Ming University, Taipei, 11221 Taiwan (ROC); 7 Institute of Neuroscience, National Yang Ming University, Taipei, 11221 Taiwan (ROC); 8 Taiwan International Graduate Program in Molecular Medicine, National Yang-Ming University and Academia Sinica, Taipei, Taiwan (ROC)

**Keywords:** Phenotypic screening, Induced pluripotent stem cells

## Abstract

Alzheimer’s disease (AD) is the most common type of dementia and also one of the leading causes of death worldwide. However, the underlying mechanisms remain unclear, and currently there is no drug treatment that can prevent or cure AD. Here, we have applied the advantages of using induced pluripotent stem cell (iPSC)-derived neurons (iNs) from AD patients, which are able to offer human-specific drug responsiveness, in order to evaluate therapeutic candidates for AD. Using approach involving an inducible neurogenin-2 transgene, we have established a robust and reproducible protocol for differentiating human iPSCs into glutamatergic neurons. The AD-iN cultures that result have mature phenotypic and physiological properties, together with AD-like biochemical features that include extracellular β-amyloid (Aβ) accumulation and Tau protein phosphorylation. By screening using a gene set enrichment analysis (GSEA) approach, *Graptopetalum paraguayense* (*GP*) has been identified as a potential therapeutic agent for AD from among a range of Chinese herbal medicines. We found that administration of a GP extract caused a significantly reduction in the AD-associated phenotypes of the iNs, including decreased levels of extracellular Aβ40 and Aβ42, as well as reduced Tau protein phosphorylation at positions Ser214 and Ser396. Additionally, the effect of GP was more prominent in AD-iNs compared to non-diseased controls. These findings provide valuable information that suggests moving extracts of GP toward drug development, either for treating AD or as a health supplement to prevent AD. Furthermore, our human iN-based platform promises to be a useful strategy when it is used for AD drug discovery.

## Introduction

Alzheimer’s disease (AD) is the most common form of age-related dementia, and is characterized by progressive memory loss and cognitive disturbance. A hallmark of AD pathology is neuronal loss in the cerebral cortex, which is accompanied by the progressive accumulation and spread of senile plaques and neurofibrillary tangles (NFTs)^1,2^. The senile plaques are extracellular deposits that are mainly composed of amyloid-β (Aβ) 1-40/1-42 peptides; these peptides are created by proteolytic cleavage of amyloid precursor protein (APP) by β-secretase, followed by γ-secretase^3,4^. The NFTs are intraneuronal aggregations of hyperphosphorylated Tau, a microtubule-associated protein involved in microtubule stabilization^5^. The causative relationship between amyloid plaques and Tau pathology in humans is as yet unclear.

Two basic types of AD have been identified; these are familial and sporadic. The familial form of AD (FAD) is rare and affects less than 5% of AD patients; it is inherited in an autosomal dominant manner and the disease manifests at an early age. Mutations in APP, *presenilin 1* (*PSEN1*) or *presenilin 2* (*PSEN2*) gene account for the majority of FAD^6^. These mutations result in incorrect cleavage of the APP protein, which produces pathological Aβ protein, especially Aβ1-42; this peptide has a greater tendency to form fibrillary amyloid deposits^7^. The genotype-to-phenotype relationship provides evidence that Aβ1-42 plays a causal role in at least some cases of AD. However, in humans, the majority of AD cases are sporadic and late-onset, and are without any known causative genetic mutation. It has been shown that polymorphism in the *Apolipoprotein E (ApoE)* gene is a strong risk factor for the development of late-onset AD^8^. Compared to individuals with an *ApoE ε3*/*ε3* genotype, the presence of one copy of the *ε4* allele increases AD risk by 2 to 3 fold, while the presence of two copies of *ε4* increases the risk by up to 12 fold^9^.

Current treatment options for AD are limited to targeting cholinergic and glutamatergic neurotransmission, and these approaches only provide a slight relief to the symptomology. Three acetyl cholinesterase inhibitors (donepezil, rivastigmine and galanthamine) have been approved for the treatment of patients with mild to moderate AD. In addition an NMDA (N-methyl-D-aspartate) receptor antagonist (memantine) is approved for the treatment of patients with moderate to severe AD. Although these drugs are being used clinically, they are only able to relieve symptoms and have no curative effect. Over the past decade, dozens of drugs and therapeutic strategies have been analyzed in order to attempt to slow or halt AD neuronal loss and AD cognitive deficiency, while still more are being investigated around the world even now. However, clinical trials for AD have regularly failed and there have been no new drug approvals for the treatment of AD since 2003^10–13^. Recently, the highly anticipated clinical trials of a drug called aducanumab, which was designed to slow down the worsening of AD by targeting Aβ, has been stopped because the trial results are unlikely to meet the trial’s primary endpoints. This disappointing news has increased scientific doubts regarding the amyloid hypothesis, namely that the deposition of fibrillar Aβ peptide is the primary cause of AD^14–16^. In these circumstances, pathological substrates, methodologies and the timing of any treatment may need to be reevaluated. Furthermore, non-amyloid approaches should also now be evaluated in more depth. It remains imperative to find a potential drug for the treatment of AD that can be used to treat the increasing number of AD patients throughout the world.

It is well established that certain natural products often possess therapeutic effects and that such natural products may have been used for the treatment and prevention of human diseases for centuries across various cultures. Moreover, it is well known that herbal medicines have a long history of being used to prevent and treat cognitive decline, one AD-like symptom^17,18^. *Graptopetalum paraguayense* (GP), a herbal medicine commonly used in Taiwan, is considered to have a range of potentially beneficial effects; these include the lowering of blood pressure and blood glucose, the alleviating of hepatic disorders, the inhibition of inflammation, anti-hepatic fibrosis activity, and anti-hepatoma activity, as well as having neuroprotective and anti-inflammatory effects on inflammation-associated neurological diseases^19–24^. In addition, partially purified fractions of GP have been found to have potential when treating chronic hepatitis B patients who have associated metabolic syndrome or liver cancer^25,26^.

The current approaches to study AD involve the use of *in vitro* cultures of rodent cells and various *in vivo* models involving transgenic mice, and these have resulted in the basis of our current mechanistic understanding of AD. However, the candidate drugs developed for AD often failed in phase 2 or 3 of their clinical trials, even after successful preclinical studies^27–29^. The cause of this low success rate during drug development may, at least partially, be attributed to differences in drug responsiveness between human beings and the model animals, to variations in the drug dosages needed and/or to various differences in the transgenes that have been used to mimic the disease condition. Thus, due to the extreme sensitivity of “*in vitro*” manipulations and the very limited availability of human mature neurons, the study of AD remains a major challenge. In the last decade, research on human induced pluripotent stem cells (hiPSCs) has created great hope in terms of their therapeutic applications to various diseases and in regenerative medicine^30^. These hiPSC-derived and differentiated cells allow researchers to study the impact of a distinct cell type on health and disease, as well as allowing the performance of therapeutic drug screening using a human genetic background.

During the present study, we established hiPSC lines from AD patients bearing the FAD mutation and bearing the ApoE ε4 polymorphism and, furthermore, differentiated these lines into their corresponding neurons (AD-iNs). This offers the opportunity to carry out human-specific drug responsiveness studies in order to evaluate the therapeutic effects of GP on AD. We found that AD-iNs exhibited notably higher levels of various pathological markers such as extracellular Aβ accumulation and Tau protein phosphorylation. Upon treatment with GP extract, these AD-related phenotypes were significantly reduced in all the AD-iNs and also in the control iNs. These findings provided valuable information and this evaluation allows us to propose moving GP extract towards development as an AD drug treatment or towards its use as health supplement to prevent AD. Furthermore, the human iN-based platform promises to be a useful strategy when exploring novel hypotheses regarding AD pathogenesis and it should be able to help with the testing of novel diagnostic methods and new therapies.

## Results

### The human induced neuron-based assay can be used for evaluation of drugs that have potential application in the prevention/treatment of AD

**Figure 1:**
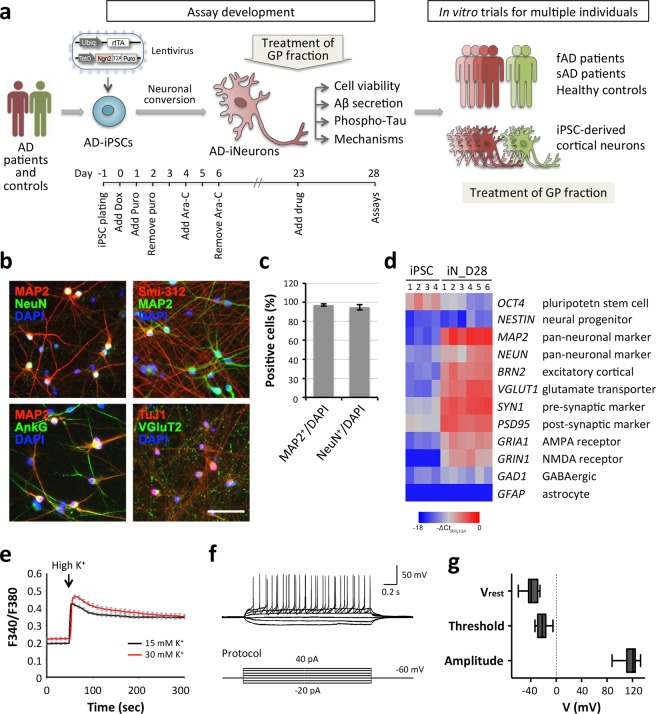
Rapid conversion of human iPSCs into neurons by forced expression of Ngn2. (**a**) Overview of the research strategy to study the effects of *Graptopetalum paraguayense (GP)* on the alleviation of various AD-associated phenotypes. Briefly, using AD patient neurons derived from iPSCs, we established a robust and reproducible assay for amyloid-β peptide (Aβ) and phosphorylated Tau (p-Tau) protein, which are two well established pathogenic molecules associated with AD. After reaching maturity, the induced neurons (iNs) were treated with GP extract and then examined for cell viability and the two AD-related markers. (**b**) Representative images of hiPSC-derived glutamatergic neurons immunolabeled for MAP2, Smi312, NeuN, Syp, PSD-95 or vGluT2 at day 28 (D28). Scale bar, 50 μm. (**c**) The differentiation efficiency is shown as MAP2^+^ or NeuN^+^ over total DAPI^+^ cells. (**d**) Heatmap of the RT-qPCR analysis of expression levels of the genes listed on the right. The mRNA was harvested from six independent batches of iNs at D28. Levels are normalized using RPL13A mRNA levels as an internal control. (**e**) Intracellular Ca^2+^ levels were measured upon K^+^ stimulations in iNs using fura-2 AM ratiometric image acquisition. Data represent mean ± SEM (n = 17–19 cells from 3 different fields for each stimulation). (**f**) Representative traces for current clamp recording at day 28. Membrane potential was held at ~−60 mV and voltage defections (mV) are shown. The multiple action potential generation (upper panel) induced by consecutive pulses of current injections (lower panel) applied with 10 pA step sizes from −20 pA to 40 pA. (**g**) Resting membrane potential (Vrest), action potential threshold (Threshold), and amplitude of action potential (Amplitude) was quantitated and shown (n = 20 cells from 6 different coverglasses).

Human neuronal cultures derived from hiPSCs have been used to create a neurological “disease in a dish” model^31^. To evaluate the therapeutic potential of target drugs on AD, we first sought to establish a human neuron-based *in vitro* model. It has been reported that hiPSCs are able to be converted into functional neuronal cells with nearly 100% yield and purity in less than 2 weeks by the forced expression of a single transcription factor Neurogenin-2 (Ngn2)^32^. As the consistent differentiation efficacy using a high purity of hiPSCs into neurons is an issue when carrying out precise modeling of a pathological condition and any subsequent drug screening/evaluation, we adopted this direct conversion technology in order to differentiate hiPSCs into cortical neurons. Briefly, hiPSCs were differentiated rapidly into neurons by inducing Ngn2 expression (Fig. 1a). Cell morphological changes during the differentiation process were monitored and are shown in Fig. S3. On day three, after changing the medium into Neurobasal supplemented with B27, brain-derived neurotrophic factor (BDNF) and neurotrophin-3 (NT-3), the cells started converting into a neuron-like morphology. Furthermore, axons and dendrites were clearly present after one week. The mature neuronal morphology appeared after less than two weeks.

To confirm these cells had a neuronal identity, we stained the hiPSC-derived neurons (iNs) for various neuronal markers at 28 days after induction. The iNs were able to display a pan-neuronal marker (NeuN), a somatodendritic marker (MAP2) and an axonal marker (Smi-312) (Fig. 1b). Most of the iN cells contained a single axonal initial segment (AIS), as detected by ankyrin G (AnkG) staining, which suggests that they were exhibiting mature polarity after 4 weeks. Importantly, all iNs expressed vesicular glutamate transporter 2 (VGluT2), a marker of glutamatergic neurons, indicating a homogeneous population of excitatory glutamatergic neurons. It should be noted that this differentiation protocol provides a means of generating iN cells that are of high purity (more than 95% of cells being iNs) as judged by the immunostaining of NeuN^+^ and MAP2^+^ cells (Fig. 1c). For a more comprehensive characterization, we used quantitative reverse transcription PCR (RT-qPCR) analysis to interrogate the expression of a series of markers in the iN cells (Fig. 1d). The iN cells barely expressed the pluripotent stem cell marker (*OCT4*) and the progenitor cell marker (*NESTIN*). Instead, there was high-level expression of two pan-neuronal genes (*MAP2* and *NEUN*), of a cortical neuronal gene (*BRN2*), of two synaptic genes (*SYN1* and *PSD95*), of an AMPA receptor gene (*GRIA1*), and of a glutamate receptor gene (*GRIN1*) in the 4-week-old iN cells, which indicates that they were fully differentiated. In addition, the glutamatergic neuronal marker (*VGLUT1*) was able to be detected in six of the independent differentiations, whereas the GABAergic marker *GAD1* and the astrocyte marker *GFAP* were exclusively found in all differentiations, which together suggest a reproducible generation of glutamatergic cortical neurons. To further verify whether iNs derived from hiPSCs are functionally active, the electrophysiological properties and cytosolic Ca^2+^ influx in response to membrane depolarization were characterized. To determine the dynamics of intracellular Ca^2+^ uptake and buffering in iNs, intracellular Ca^2+^ measurement was performed using live single-cell fura-2 Ca^2+^ imaging analysis. The iNs exhibited an elevation in 15 mM and 30 mM K^+^-evoked Ca^2+^ influx, indicating the presence of functional voltage-gated Ca^2+^ channels on the plasma membrane of iNs (Fig. 1e). Mature and functional neurons are also identified by their ability to exhibit action potential. At day 28, whole-cell patch-clamp recording revealed that most hiPSC-derived neurons (95%, n = 20 cells) generated action potentials. These neurons showed a resting potential of −38.16 ± 2.20 mV and a membrane resistance of 57.83 ± 8.67 MΩ, and exhibited action potential firing threshold and amplitudes at −21.55 ± 1.78 mV and 116.97 ± 2.46 mV, respectively (Fig. 1f,g). Taken together, iNs display stimulus-induced channel activities and electrical properties. Moreover, these results demonstrate that the iNs are able to be cultured at high purity and with low batch-to-batch variability, making them suitable for the evaluation of drug candidates.

### Generation and characterization of iNs with FAD and SAD mutations

**Figure 2:**
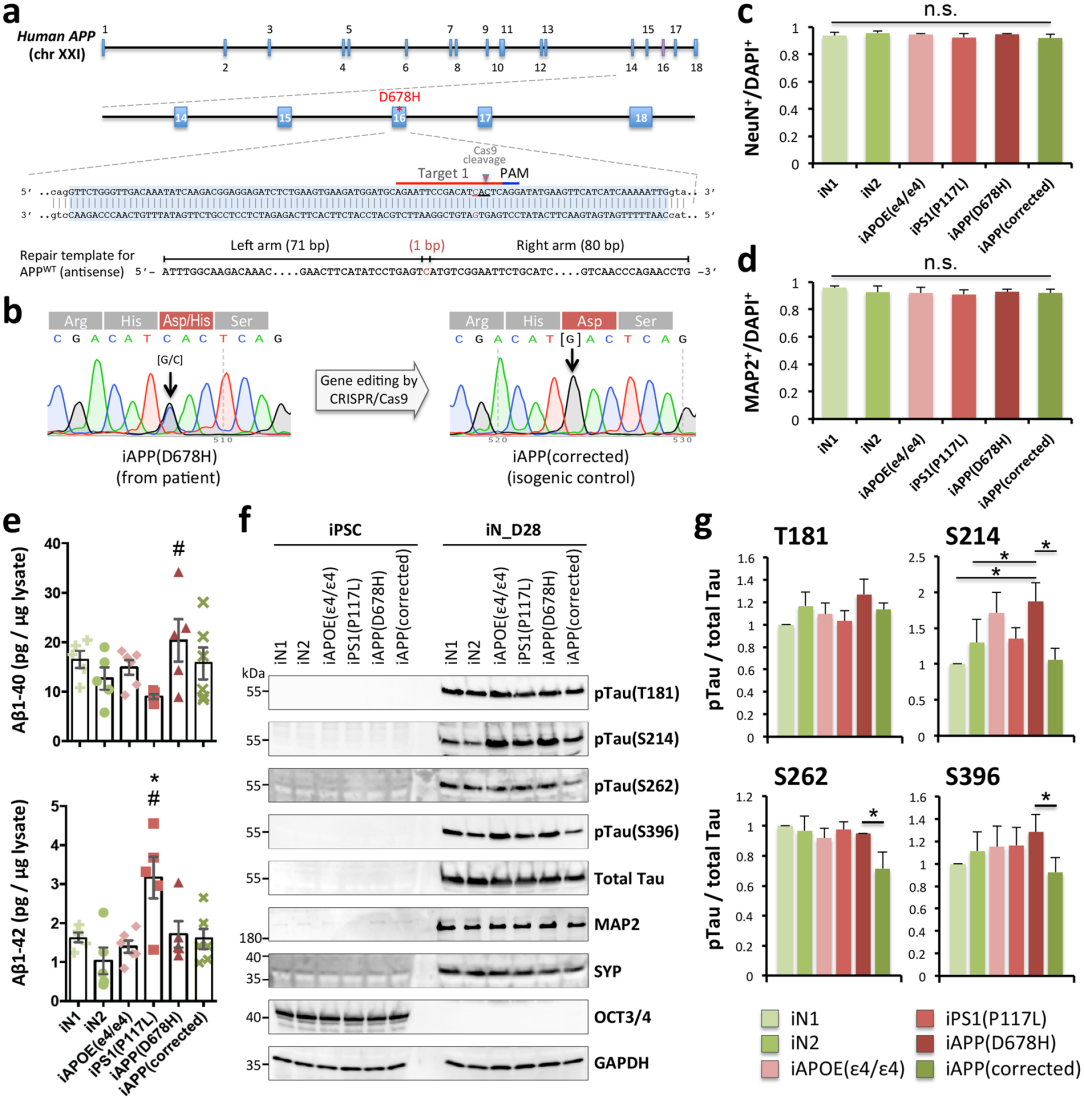
Characterization of the AD-associated phenotypes of the AD-iPSC derived neurons. (**a**) The strategy of genome editing for human APP exon 16 using CRISPR/Cas9 system. The ssODN donor used for homology-directed repair (HDR) is shown below. (**b**) The corrected iPSC clone was confirmed by Sanger sequencing. (**c**,**d**) The differentiation efficiency of the various iPSC lines is shown as NeuN^+^ (**c**) or MAP2^+^ (**d**) over total DAPI^+^ cells. There is no statistically significant difference between groups as determined by one-way ANOVA. (**e**) ELISA quantification of extracellular Aβ1–40 and Aβ1-42 of iNs at D28. Data represent mean ± SEM (n = 5~6 independent batches of differentiation for each line; *p < 0.05 and ^#^*p* < 0.05 by one-way ANOVA and Tukey’s *post hoc* test for multiple comparisons for iN1 and iN2, respectively). (**f**) Western blotting analysis was used to monitor the expression of Tau phosphorylated at T181, S214, S262 and S396 and total Tau in the control and AD-iPSC derived neurons at D28. GAPDH was used to confirm that there were similar protein loadings across samples. (**g**) Quantitative results of (**f**). The intensity of the p-Tau signals was normalized against total Tau. Data represent mean ± SEM (n = 3-4 independent batches of differentiation for each line; *p < 0.05 by one-way ANOVA and Fisher’s LSD test for multiple comparisons).

To confirm that the system developed in this study is able to recapitulate the various AD-associated phenotypes, including extracellular Aβ accumulation and Tau protein phosphorylation, we compared the iNs derived from AD patients with APP or PSEN1 mutations or with the ApoE ε4 polymorphism to those from normal subjects (Table 1). Moreover, we also corrected the FAD APP_D678H mutation using clustered regularly interspaced short palindromic repeats (CRISPR)/Cas9 technology^33^ (Fig. 2a). We were able to thus generate a gene-corrected isogenic clone iAPP(corrected), where the single-base C mutation (D678H) in the *APP* gene has been replaced with a G base. The successful exchange was validated by Sanger sequencing (Fig. 2b). We have furthermore confirmed that the other DNA sequences had remained intact and that no frameshift or other mutation had been introduced into the gene-edited site; this was done by analyzing the region around the CRISPR cutting site (Fig. S2).

**Table 1:** Human iPSC lines.

Lines	Gender	Age at biopsy	Genotype*	APOE status	Clinical diagnosis at time of biopsy
iN1	Female	45	WT	ε3/ε3	Not determined
iN2	Male	52	WT	ε3/ε4	Not determined
iAPOE(ε4/ε4)	Female	66	WT	ε4/ε4	AD with progressive memory impairment for many years
iPS1(P117L)	Male	42	PS1(P117L)	ε3/ε3	AD with progressive memory impairment for many years
iAPP(D678H)	Female	63	APP(D678H)	ε3/ε4	AD with progressive memory impairment for many years
iAPP(corrected)	Female		APP(D678D)	ε3/ε4	A CRISPR/Cas9-corrected line

*Exome sequencing of APP, PSEN1 and PSEN2 genes.

Next, to analyze the functional aspects of AD, we investigated Aβ secretion from hiPSCs or iNs of six lines, three lines derived from AD patients, two from controls and one APP-corrected line (Table 1), simultaneously. All hiPSCs exhibited the typical characteristics of pluripotent stem cells, namely similar morphology to embryonic stem cells and the presence of pluripotency markers (see Figs. S1 and S5). The efficiency of iN cell conversion is high (91% to 96%) and reproducible for the various hiPSCs, and revealed no significant difference in the ability to generate iNs when the various different AD mutations were present (Figs. 2c,d and S4). Furthermore, iN cells displayed healthy morphologies in cell body and neurites across all lines (Figs. S3, S4 and S6). The conditioned medium from the control and patient-derived hiPSCs was very low; this meant that it was not possible to compare the production and secretion of Aβ1-42 and Aβ1-40 across the hiPSC lines. However, the Aβ secretion in the conditioned medium from the iNs was greatly increased and was measurable, indicating that Aβ secretion undergoes significant changes during neuronal differentiation. The secretion of Aβ1-40, the most abundant Aβ isoform, and of Aβ1-42, the more aggregation-prone and toxic isoform in nature was then determined in iNs. The amounts of extracellular Aβ1-40 in iAPP(D678H) were significantly increased compared to iN2. However, the extracellular Aβ1-42 was remarkably elevated in iPS1(P117L), but not in iAPP(D678H), compared to normal lines. No significant differences in either Aβ1-40 or Aβ1-42 were detected in iAPOE(ε4/ε4) and iAPP(corrected). The results indicated that these iN cells with the PS1 or APP mutation, which are linked to FAD, secrete significantly more Aβ1-42 or Aβ1-40, respectively (Fig. 2e).

To explore other key pathological events in AD, we investigated whether iN cells exhibit abnormal phosphorylation of the Tau proteins by Western blotting. As shown in Fig. 2f and g, the Tau protein was markedly hyper-phosphorylated at S214 in the iAPP(D678H)-derived neurons compared with the controls, iN1 and iN2. Notably, we found ~1.4 fold higher levels of phosphorylated Tau (p-Tau) protein at positions S214, S262 and S396 in the iAPP(D678H)-derived neurons, which contains a heterozygous WT/D678H mutation, compared to the isogenic WT/WT control iAPP(corrected) (Fig. 2g). These results demonstrate that there is recapitulation in the present hiPSC-neuron model not only of Aβ accumulation in these cells, but also of the tauopathy that is associated with AD.

Lowering Aβ and p-Tau levels has been proposed as a key therapeutic opportunity in AD treatment. To our knowledge, there is no other *in vitro* platform that targets endogenously both Aβ and p-Tau in post-mitotic human neurons that can be used for AD drug discovery. As our AD-iN cultures showed AD-associated features, including extracellular Aβ accumulation and Tau protein phosphorylation, they are able to act as an ideal system for evaluating potential therapeutic agents targeting the prevention/treatment of AD.

### GP has been predicted to be a potential therapeutic agent for Alzheimer’s disease by using GSEA

Chinese herbal medicines (CHMs) have been suggested to have the potential to ameliorate AD progression, perhaps by partially improving cognitive functioning. To identify potential therapeutic agents for AD from among CHMs, we conducted a systematic review and established a set of gene signatures obtained from AD patients based on eight publications (Table S1)^34–41^. Moreover, we also applied L1000 microarray profiling^42^ to generate the 11,641 molecular signatures of differentially expressed genes from human cancer cell lines, including neuroblastoma SH-SY5Y cells, when they had been one by one treated with more than 500 different CHMs^43^. We next employed eight AD-related gene expression profiles as inputs to query 11,641 L1000-based gene expression profiles using gene set enrichment analysis (GSEA). GSEA is a computational method that determines whether an *a priori* defined set of genes shows statistically significant differences between two biological states. We were able to identify a group of CHMs that have the potential to reverse the gene signature of AD patients (Table 2). This analysis raised the possibility that targeting these AD-associated gene signatures using a specific CHM could result in amelioration of the various AD phenotypes. Notably, *Graptopetalum paraguayense* (GP) was one of the top-ranking candidates among the CHMs and this CHM showed a strong negative correlation with the AD signatures compared with some other promising herbs for AD therapy, such as *Scutellaria baicalensis* and *Curcuma wenyujin*^44,45^.

**Table 2:** Chinese Herbal Medicines that have potential For the treatment of AD as predicted by gene set enrichment analysis (GSEA).

Herb name	ES	NES	NOM *p*-value	FDR q-value
*Graptopetalum paraguayense*	−0.541	−2.007	<0.001	0.001
*Polygonum cuspidatum*	−0.501	−1.833	<0.001	0.007
*Da Chai Hu Tang*	−0.473	−1.737	<0.001	0.019
*Scutellaria Baicalensis*	−0.407	−1.516	0.008	0.088
*Polygoni Multiflori Caulis*	−0.389	−1.431	0.030	0.150
*Curcuma wenyujin*	−0.351	−1.316	0.079	0.289

ES, enrichment score; NES, normalized ES; NOM, normal; FDR, false discovery rate.

### Effects of a GP extract on the accumulation of extracellular Aβ in AD-iNs

**Figure 3:**
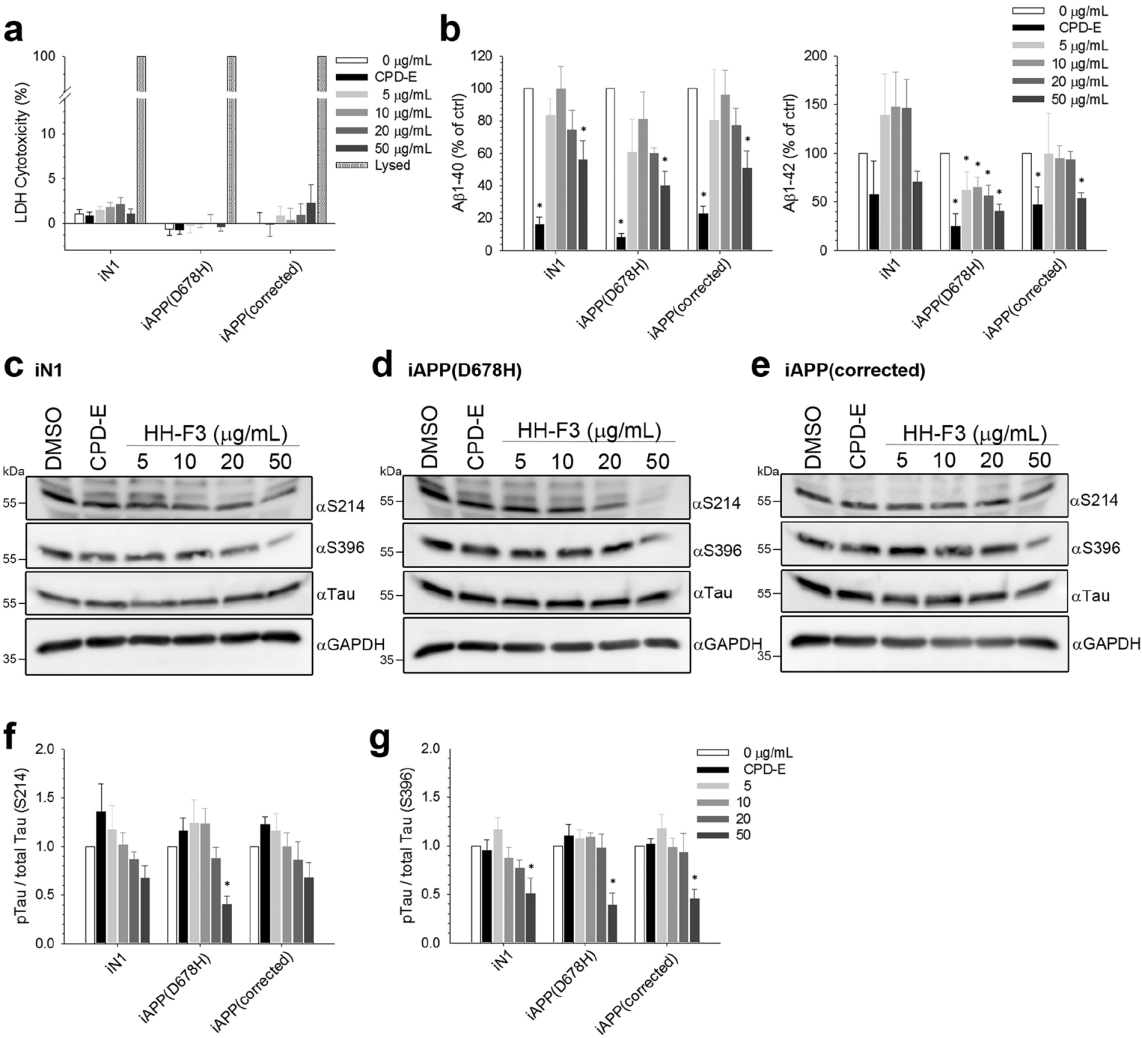
The dosage effects of GP extract on the secretion of Aβ and phosphorylation of Tau protein in iPSC-derived neurons. (**a**) iN1, iAPP(D678H) and iAPP(corrected) derived neurons were exposed to 5, 10, 20 and 50 μg/mL of HH-F3 for 5 days and the effects on cell viability were tested by determining intracellular glycolytic enzyme activity. LDH released into medium was measured and compared to untreated cells (low control) and lysed cells (high control). Values are represented as mean ± SEM; n = 4~5 independent batches of differentiation. (**b**) The dose effect of HH-F3 on the human Aβ1-40 and Aβ1-42 concentrations using ELISA assays was measured and then normalized to control. Treatment with CPD-E was used a positive control. The results are represented as mean ± SEM; n = 4 independent batches of differentiation. *p < 0.05 by one-way ANOVA with Fisher’s least significant difference method. (**c**–**e**) Western blotting analysis was used to validate the level of p-Tau phosphorylated at Ser214 and Ser396, as well as total Tau in control, iAPP(D678H) and iAPP(corrected)-derived neurons after treatment with various concentrations of HH-F3 for 5 days. (**f**,**g**) The densitometric signal for each sample was adjusted using total Tau, and the ratio of treated sample to control sample was calculated for each cell line. The quantitative results are shown as means ± SEM; n = 5~7 independent batches of differentiation. *p < 0.05 by one-way ANOVA with Fisher’s least significant difference method.

An extract of *Graptopetalum paraguayense*, designated HH-F3, was obtained that was enriched in active ingredients. HH-F3 also exhibited anti-cancer properties and inhibited tumor growth^25^. To investigate the effects of HH-F3 on the alleviation of AD-related phenotypes in AD-iN cells in terms of a decrease in Aβ accumulation and lower Tau phosphorylation, we differentiated iAPP(D678H) into glutamatergic neurons. After 23^rd^ days of differentiation, the mature iNs were treated with HH-F3 at dosages of 5, 10, 20 and 50 μg/mL. After five-days of treatment, the toxicity of various doses of HH-F3 was assessed by cell viability measurements using the lactate dehydrogenase (LDH) assay. We found none of treatment dosages induced a significant increase in the LDH response of the cells, indicating that there was little toxicity derived from HH-F3 during the exposure period (Fig. 3a). Meanwhile, conditioned medium was harvested to allow quantification of extracellular Aβ1-40 and Aβ1-42 by ELISA. We found that administration of HH-F3 reduced extracellular Aβ1-40 at a dosage of 50 μg/mL, while at the same time markedly decreasing Aβ1-42 in a dose-dependent manner when these iAPP(D678H)-derived neurons were used (Figs. 3b and S7). HH-F3 treatment also decreased extracellular Aβ1-40 released by iN1 and iAPP(corrected) derived neurons; however, it did not affect extracellular Aβ1-42 released by iN1 and iAPP(corrected) at the lower concentrations. Treatment with compound E (CPD-E), a potent inhibitor of γ-secretase as a positive control, significantly attenuated the release of both Aβ1-40 and Aβ1-42 in these 3 lines. It is noted that the reduction of secreted Aβ1-42 in iN1 caused by CPD-E treatment did not reach statistical significance (Fig. 3b), which may due to the variability between differentiations (Fig. S7). Together, these findings suggest that this GP fraction reduces both extracellular Aβ1-40 and Aβ1-42 accumulation at dosages that are non-toxic to the AD-iNs.

### Effect of the GP fraction on phosphorylation of Tau protein using AD-iNs

Lowering Tau levels have also emerged as a key therapeutic opportunity for the treatment of AD. To determine whether HH-F3 treatment is able to decrease Tau phosphorylation in AD-iNs, we measured using Western blotting the amount of p-Tau relative to total Tau level in lysates from iNs derived from AD patients (Fig. 3c–e). After five-days of HH-F3 exposure at various concentrations (5-50 μg/mL), protein extracts of iAPP(D678H) were subjected to Western blotting using antibodies against total Tau protein and p-Tau phosphorylated at positions Ser214 and Ser396; from these results the ratios of p-Tau to total Tau were calculated. The quantitative results revealed that 50 μg/mL HH-F3 significantly reduced Tau phosphorylation by 40% at Ser214 using iAPP(D678H) neurons, while HH-F3 showed no statistically significant effects on either iN1 or iAPP(corrected) neurons (Fig. 3f). The effect of HH-F3 was more remarkable in terms of decreases in the level of p-Tau phosphorylated at Ser396, which were 49%, 61% and 55% upon treatment with 50 μg/mL of HH-F3 using iAPP(D678H), iAPP(corrected) and iN1 cells, respectively (Fig. 3g). However, HH-F3 showed no influence on the phosphorylation of p-Tau at either Ser262 or Thr181 (see Fig. S8). Taken together, our results showed that the GP extract was able to lower, in iNs with an APP mutation, the accumulation of extracellular Aβ peptides and the phosphorylation of Tau protein at Ser214/Ser396, which are the two main pathological markers of AD.

### The effects of GP extract on iN cells from multiple AD patients

**Figure 4:**
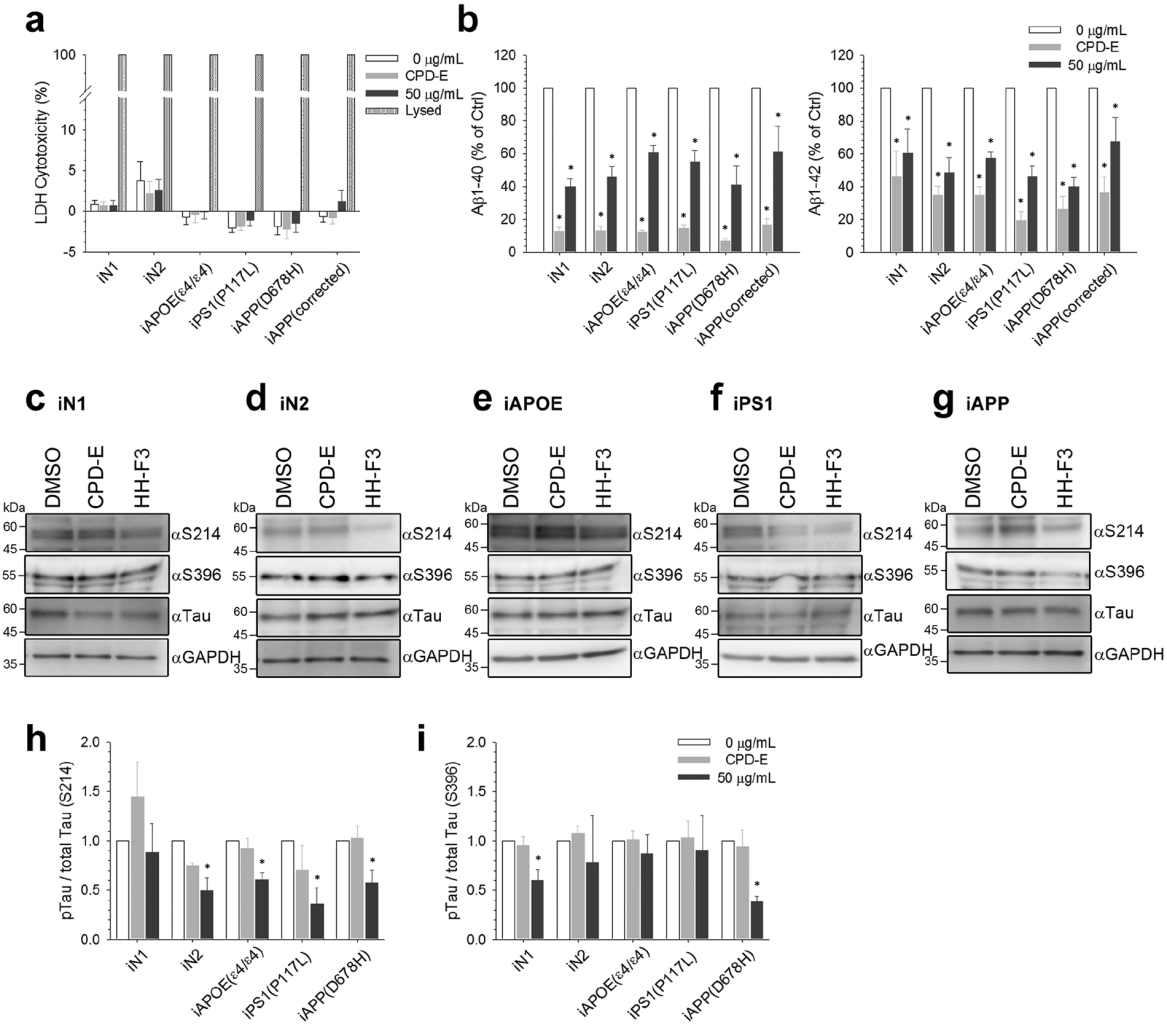
The effects of GP extract on the secretion of Aβ and phosphorylation of Tau proteins in iNs from multiple AD patients carrying different mutations. To elucidate the effect of HH-F3 on an expanded population of AD patients, iN2, iPS1(P117L) and iAPOE(ε4/ε4)-derived neurons were added into the assessment. Cells were treated with 50 μg/mL for 5 days. (**a**) HH-F3 cytotoxicity was examined by LDH assay and the results are shown as mean ± SEM; n = 5~7 independent batches of differentiation. (**b**) The concentrations of secreted Aβ1-40 and Aβ1-42 in the medium were measured by ELISA assays after administration of HH-F3 and normalized to control in each line. The results are represented as mean ± SEM; n = 5~6 independent batches of differentiation. *p < 0.05 by one-way ANOVA with Fisher’s least significant difference method. (**c**–**g**) Representative Western blotting results against p-Tau at Ser214 and Ser396 in iN1, iN2, iAPOE(ε4/ε4), iPS1(P117L) and iAPP(D678H)-derived neurons after HH-F3 treatments are shown. (**h**,**i**) The intensity of p-Tau signals was normalized against total Tau as a control, and the quantitative results are shown as mean ± SEM; n = 3~9 independent batches of differentiation. *p < 0.05 by one-way ANOVA with Fisher’s least significant difference method.

Up to this point, the analysis was conducted using only one FAD-iN with the APP(D678H) mutation, which was compared to two control lines. To survey further the response to GP when an expanded population of AD derived cell lines was used, we conducted the same studies using hiPSCs from multiple AD patients. We carried out this evaluation by using one additional hiPSC with the AD-causative mutation P117L in PSEN1 gene, one sporadic AD-iPSC with the ApoE ε4/ε4 polymorphism, and one more control hiPSC (iN2) (Table 1). At 23^rd^ days of differentiation, the various iN cultures were separately treated with HH-F3 at dosages of 50 μg/mL for five days. The treatment of HH-F3 successfully brought about between 30% and 60% reduction in both Aβ1-40 and Aβ1-42 levels in all iN lines (Figs. 4b and S7), while no dramatic cytotoxicity was observed by LDH assay (Fig. 4a). The intact neuronal morphologies of all iN lines after treatment of 50 μg/mL HH-F3 further revealed non-toxic effects of indicated concentrations of HH-F3 (see Fig. S6).

To examine whether HH-F3 treatment is also able to decrease Tau phosphorylation in AD-iNs from different individuals, we determined the amount of p-Tau relative to total Tau levels in the lysates from the iNs of the three AD patients and two controls (Fig. 4c–g). After five-days of HH-F3 exposure, protein extracts were subjected to Western blotting using antibodies against total Tau protein and p-Tau at positions Ser214, Ser396, Ser262 and Thr181, and then the ratios of p-Tau over total Tau were calculated. We found that 50 μg/mL of HH-F3 significantly reduced Tau phosphorylation at S214 in all examined AD-iNs, iAPOE(ε4/ε4), iPS1(P117L) and iAPP(D678H), as well as in one control line iN2 (Fig. 4h). HH-F3 treatment also remarkably decreased the phosphorylation of Tau at S396 in iAPP(D678H) and iN1 neurons (Fig. 4i). In addition, the hyper-phosphorylation of Tau at S262 in iPS1(P117L) was significantly attenuated but that at Thr181 in iAPOE(ε4/ε4) was found to be slightly increased (see Fig. S8). The results of pTau/total Tau measurements in AD-iNs mirrored the Aβ findings. Namely that administration of HH-F3 notably lessened both extracellular Aβ1-40 and Aβ1-42 accumulation as well as lowering the pTau/total Tau ratios from these AD-iNs. Memantine is a medication that has been approved by United States Food and Drug Administration (FDA) for the treatment of moderate-to-severe AD. It is an amantadine derivative with low to moderate-affinity for N-methyl-D-aspartate (NMDA) receptors and inhibits the prolonged influx of Ca^2+^ ions into cells^46^. In contrast to the above results for treatment with the GP extract, treatment with memantine did not result in any effect in our AD-iN model system on the amount of Aβ released or p-Tau levels of the cells (see Fig. S9). From these results, we are able to highlight GP as a new potential drug for the prevention/treatment of AD.

## Discussion

In this study, we generated three lines of hiPSCs from AD patients and one APP-corrected line; using these we have established a robust protocol to differentiate hiPSCs into mature glutamatergic neurons (Fig. 1). Our results reveal that the elevation of extracellular Aβ in the iPS1(P117L) and iAPP(D678H) derived neurons and the hyper-phosphorylation of Tau at Ser214 in the iAD-derived neurons compared to the control neurons (Fig. 2), suggest a recapitulation of these AD phenotypes in the AD-iPSC derived neurons. GP, which has been predicted to be a potential herbal medicine that can be used for AD therapy by GSEA, was evaluated for its anti-AD effects using the AD-iNs from multiple patients (Figs. 3 and 4). The secretion of Aβ1-40 and Aβ1-42 was significantly reduced in all the iAD-derived neurons and also the control neurons after treatment with HH-F3, one of GP fractions enriched with active ingredients, using incubation for 5 days at a concentration of 50 μg/mL. HH-F3 treatment also decreased the hyper-phosphorylation of Tau protein at Ser214 in all iAD-derived neurons and in iN2 neurons, at Ser396 in iAPP(D678H) neurons, as well as at Ser262 in iPS1(P117L)-derived neurons. These findings suggest that this GP extract is a potential target for drug development or could be used as a healthy supplement as part of AD therapy or prevention.

The hiPSC-derived neurons hold promise when trying to analyze complex neurological diseases such as AD. Indeed, the secretion of Aβ and the hyperphosphorylation of Tau are both increased in the iPS1(P117L) and iAPP(D678H) derived neurons (Fig. 2). Nevertheless, the levels of Aβ1-40 and Aβ1-42 was not elevated across all AD lines (Fig. 2e). The fact of this phenomenon is likely resulted from two reasons. First, the genetic backgrounds of all lines are different. Previous study has shown that APP processing, Aβ metabolism and deposition vary by genetic background in mouse, leading to alterations in steady-state levels of Aβ^47^. It was also reported that up to 80% of AD cases are hereditary base on human pedigree study^48^ and genetic alterations are possibly to modify the production of pathogenic Aβ^49^. These hiPSC lines also possess different APOE status, which may modulate the APP cleavage^50^. Second, our recent unpublished data revealed an elevated level of Aβ1-42 in more aged, at around day 50, in iN cells carrying APP(D648H) mutation. In addition, the gene expression, as well as the length of axons and dendrites, showed no differences when the normal iNs and the AD-iNs were compared (see Figs. S4 and S5), which indicates that these neurons may only mimic the AD phenotypes at the pre-clinical or early stage of the disease. It has been shown that hiPSCs obtained from patients with late-onset diseases fail to show their age-associated markers during the reprogramming process^51^. The findings demonstrate AD phenotypes may be more appearing in more aged neurons. Similarly, genetic background^52,53^ and the age of iN cells may be the causes for the results of Tau phosphorylation (Fig. 2g). However, the hiPSC-derived neurons at day 28 are functionally mature in terms of excitability, as well as neuronal gene and protein expression (Figs. 1 and 2, S4 and S5), suggesting these neurons at day 28 are functionally suitable to evaluate drug effects. In the future, aging factors need to be considered and embedded into the current platform. Additionally, a three-dimensional tissue engineering approach^54,55^ needs to be developed using the current method of differentiating hiPSCs in order to better model AD by fully recapitulating the pathology of AD, including Aβ deposits and neurofibrillary tangles^56–58^.

The amyloid hypothesis proposes that the accumulation of Aβ develops into senile plaque and this is the main cause of neurotoxicity and Tau pathology; this then leads to neuronal death and neurodegeneration^15,59^. This theory has had some doubts raised about it recently because several drugs that effectively remove the accumulation of Aβ have ended up failing to improve cognitive functions when used in clinical trials; however, this hypothesis has dominated research and drug development for over 25 years^60,61^. We have found that HH-F3 treatment does significantly reduce the extracellular level of Aβ1-40 and Aβ1-42 in the normal lines too, as well as in the three AD-iN lines (Figs. 3b and 4b); furthermore, the treatment had a greater effect on the secretion of Aβ1-42 in iAPP(D678H) at lower dosages (Fig. 3b). Additionally, HH-F3 was demonstrated here to attenuate the hyper-phosphorylation of Tau proteins at Ser214 in all AD-iNs, including iAPOE(ε4/ε4), iPS1(P117L) and iAPP(D678H), and a control iN2. HH-F3 was also shown to diminish phosphorylation of Tau at Ser396 in iAPP(D678H), iN1 and iAPP(corrected) (Fig. 3g), but not in the other iAD-derived neurons (Fig. 4i). This suggests that HH-F3 may have a more striking benefit on patients who carry the APP mutation at D678H. After numerous failures of the amyloid hypothesis, Tau-targeted potential therapies have gained more attention recently. The Tau hypothesis emphasizes that the principle causative substance of AD is Tau^62^, and previous studies have revealed that Tau pathology, as measured by positron-emission tomography (PET) imaging, is closely associated with AD progression^63^. Regarding the decrease in extracellular Aβ release and the hyperphosphorylation of Tau proteins, HH-F3 may be a potential therapeutic drug for both these aspects of AD.

Abundant evidence suggests that oxidative stress may be involved in the neuronal death induced by Aβ toxicity and antioxidants have been reported to protect neurons from this damage^64–66^. In addition, Aβ may play a key role in driving neuroinflammatory responses that result in the secretion of proinflammaroty cytokines^67^ and sustained overexpression of inflammatory cytokines seems to exacerbate Tau pathology^68^. It has been demonstrated that water-based extracts of GP have anti-oxidative and anti-inflammatory properties against CCl_4_-induced oxidative damage using hepatocytes^23^. A previous study has also indicated that an ethyl ether extract of GP possesses neuroprotective and anti-inflammatory activities regarding inflammation-associated neurological diseases^24^. These findings point toward GP as a potential therapeutic strategy that may include targeting of microglia activation, control of oxidative stress, and inhibition of iNOS expression, all of which are known to reduce ischemic brain injury, and imply that GP extracts represent a valuable source for the development of neuroprotective agents. However, our result showed no significant change on the level of reactive oxygen species in iNs after GP treatment (see Fig. S10). Our present results further suggests that HH-F3 may be a potential therapeutic drug for AD and act in AD-iNs by reducing the extracellular level of Aβ1-40 and Aβ1-42, and by attenuating the hyper-phosphorylation of Tau proteins at Ser214.

Our understanding of AD and various related predictive preclinical models has been mostly predicated on studies using rodent models, and these have resulted in our current mechanistic knowledge of the disease; this may be related in turn to the extremely high failing rate of recent clinical trials^29,61^. In the present study, hiPSCs are able to be rapidly differentiated into mature neurons with high purity. The scale and high yield suggests this model is suitable for assessment of potential drugs for the treatment/prevention of AD (Fig. 1) instead of high capacity screening. We utilized hiPSC-derived neurons and a genome editing technology approach to decipher the potential therapeutic effects of HH-F3 on the release of Aβ and the hyperphosphorylation of Tau (Figs. 3 and 4), which are the hallmarks of AD pathology. The hiPSCs-derived neurons have proved useful in these studies, which were aimed at elucidating the pathogenesis of AD using human neurons. These iNs also can serve as a predictive preclinical model on a human genetic background and thus provide an model for translating between animal models and humans, which helps to bridge a critical niche gap in research.

## Methods

### Generation and culture of human induced pluripotent stem cells (hiPSCs)

Patients with AD were evaluated and tracked; their blood samples or skin biopsy were then collected by neurologists at Taipei Veterans General Hospital, Taiwan. The AD biopsy samples of these patients were reprogrammed into iPSCs using Sendai viral transduction (CytoTune-iPS Sendai Reprogramming kit, Thermo Fisher Scientific, USA), which involves four transcription factors, namely human octamer-binding transcription factor (OCT)3/4, sex determining region Y-box 2 (SOX2), Krüppel-like factor 4 (KLF4) and cellular myelocytomatosis (cMYC). The establishment of the AD-hiPSC lines followed the Policy Instructions of the Ethics of Human Embryo and Embryonic Stem Cell Research in Taiwan. In addition, approval from the Institutional Review Boards of Taipei Veterans General Hospital and National Yang-Ming University were obtained. Informed consent was obtained from each patient’s surrogate. The successful hiPSC clones exhibited the typical characteristics of pluripotent stem cells and their differentiation ability was confirmed (Fig. S1). The control hiPSC lines, NTUH-iPSC-01-05 and NTUH-iPSC-02-02 (abbreviated to N1 and N2, respectively), were purchased from Bioresource Collection and Research Center of Food Industry Research and Development Institute, Taiwan.

hiPSC lines were routinely maintained in Essential 8 medium (Invitrogen, USA) on cell culture dishes coated with 0.5 μg/cm^2^ recombinant human vitronectin (Invitrogen). In order to passage the hiPSCs, the cells were washed twice with sterilized Dulbecco’s phosphate buffered saline (DPBS) (Invitrogen) without calcium and magnesium, and then incubated with DPBS/EDTA (0.5 mM UltraPure EDTA in DPBS) at 37 °C for three minutes. When the cells began to separate and round up, the DPBS/EDTA was removed and the cells were swiftly removed from the vessel by washing. An appropriate number of cells was then transferred to a new culture dish and maintained in a 37 °C and 5% CO_2_ incubator. Generally, the cells were sub-cultured every 4–5 days and this involved reseeding at a ratio of between 1:5 and 1:10. The culture medium was refreshed daily.

### Generation of isogenic hiPSC Lines by CRISPR/Cas9 Method

The DNA oligonucleotides used for the gRNA targeting were designed using the GeneArt CRISPR gRNA Design Tool (Thermo Fisher Scientific). To examine the cleavage efficiency of gRNA, a series of gRNAs flanking the target site were designed and synthesized. Each individual gRNA was combined with CRISPR associated protein 9 (Cas9) nuclease (Invitrogen) to form Cas9 protein/gRNA ribonucleoprotein complex (Cas9 RNP). The Cas9 RNP was then used to transfect hiPSCs via Neon Transfection System (Invitrogen). The genomic editing efficiency was evaluated by T7 endonuclease I (T7E1) assay at 48 hr post transfection. The gRNAs with highest cleavage efficiencies, and that were also in close proximity to the target site, were selected for the subsequent genome editing. For precise genome editing, the Cas9 RNPs and repair template (ssODN from IDT, Coralville, USA) were co-electroporated into hiPSCs. The transfected hiPSCs were then clonally expanded to derive isogenic cell lines. Each single-nucleotide substitution was screened using a TaqMan SNP genotyping assay (Applied Biosystems, USA) and further confirmed by Sanger sequencing.

### Lentivirus production and infection

The lentiviral vector pTet-O-Ngn2-puro was a gift from Marius Wernig (Addgene plasmid # 52047). For lentivirus production, Human Embryonic Kidney (HEK) 293 T cells were seeded at 5 × 10^6^ cells in a 10-cm dish and incubated overnight. The cells were then co-transfected with 6 μg pTet-O-Ngn2-puro or pFUW-rtTA together with 5 μg of packaging plasmid pCMV-Δ8.91 and 1 μg of envelope plasmid pVSV-G using Lipofectamine 3000 (Invitrogen) by following the manufacturer’s instructions. The supernatants were collected at 24 and 72 hours after transfection, filtered through a 0.45-μm filter to remove cell debris and then purified using a Lenti-X Maxi purification kit (TaKaRa, USA). Each virus concentrate was then separated into 100 μl aliquotes and stored at −80 °C until use. For hiPSC transduction, approximately 2 × 10^4^ hiPSCs in individual wells of a 24-well plate were infected with lentivirus containing rtTA. On the next day, the medium was replaced with fresh E8 including lentivirus containing pTet-O-Ngn2-puro. The lentivirus-infected hiPSCs were passaged and expanded for three days after transduction to allow neuronal differentiation.

### Generation of induced neurons (iNs) from hiPSCs

hiPSCs were treated with 0.5 mM EDTA and plated as dissociated cells in Essential 8 medium containing 5 μM Y-27632 (MedChemExpress, USA) at a density of 10^5^ cells/mL onto vitronectin-coated dishes on day −1. On day 0, the culture medium was replaced with Dulbecco’s Modified Eagle Medium (DMEM)/F12 containing N-2 supplement, non-essential amino acids (NEAA), human brain-derived neurotrophic factor (BDNF) (10 μg/L, PeproTech, USA), human neurotrophin-3 (NT-3) (10 μg/L, PeproTech), mouse laminin (0.2 mg/L, Invitrogen) and doxycycline (2 mg/L). On day 1, a 24 hr puromycin selection (1 mg/L) period was started. On day 2, the transfected cells were replated in Neurobasal medium supplemented with B27/Glutamax (Invitrogen) containing BDNF and NT-3. On day 5, Ara-C (2 μM, Sigma-Aldrich, USA) was added to the medium for 24 hr to inhibit the proliferation of undifferentiated cells. After this, half of the medium in each dish was replaced every 3–4 days. The iN cells were assayed on day 28 during most experiments.

### Gene set enrichment analysis (GSEA)

Gene expression profiles were obtained from cancer cells treated with small molecules and Chinese herbal medicines, in triplicate for 6 hours, followed by L1000 expression profiling^42^ by Genometry Inc. as described previously^43^. Gene set enrichment analysis (GSEA) is a powerful tool for inspecting whether identified differentially expressed genes are significantly associated with a prior biological knowledge category, and this process is widely used in genomic analysis. GSEA assigns an enrichment score equivalent to the Kolmogorov-Smirnow statistic for each predefined gene set and subsequently normalized the score according to its size. Finally, based on the normalized enrichment score, a permutation-based false discovery rate (FDR) is generated to indicate the significance of enriched gene sets. The GSEA software was downloaded from the web site http://www.broadinstitute.org/gsea/index.jsp.

### Preparation of partial purified fraction HH-F3

*Graptopetalum paraguayense (GP)* were provided by the Development Center for Biotechnology. GP leaves were ground and lyophilized into a powder at −20 °C and stored in a moisture buster at 25 °C before extraction. Next the lyophilized GP powder (15 g) was vortexed with 100% methanol (100 mL) for 5 min and centrifuged at 1,500 × g for 5 min. The supernatant was then removed and various extracts were prepared after re-suspending the pellets in 10 mL of 30% dimethyl sulfoxide (DMSO). The supernatant was then fractionated into four fractions (F1–F4) using a Sephadex LH-20 (GE Healthcare Bio-Sciences AB, Uppsala, Sweden) column. The F3 fraction (referred to as the HH-F3 fraction) was identified to be the active fraction^25^.

### Extracellular Aβ1-40 and Aβ1-42 detection

iNs were treated with GP extract for 5 days. The conditioned medium was harvested and centrifuged in the presence of the protease inhibitor cocktail plus 4-(2-aminoethyl)benzenesulfonyl fluoride hydrochloride (AEBSF, Sigma-Aldrich) and stored at −80 °C until analysis. Aβ accumulation was measured via a sensitive fluorescence based sandwich enzyme-linked immunosorbent assay (ELISA) that used a kit (Invitrogen). The detailed procedure was performed according to the manufacturer’s protocol. Neuronal Aβ levels were normalized against the total amount of protein present as determined by Bio-Rad protein assay (Hercules, USA).

### Protein extraction and western blotting analysis

Transfected cells were washed once with PBS, and then lysed with radioimmunoprecipitation assay buffer (RIPA) lysis buffer (20 mM HEPES**-**NaOH, pH 7.8, 150 mM NaCl, 1 mM EDTA, 0.1% Triton X**-**100, 50 mM NaF and 1 mM dithiothreitol) containing protease inhibitor cocktail (Roche, USA) and PhosSTOP (Roche). The cell lysates were cleared by spinning in a microcentrifuge at 20,000 × g for 10 min, and then the supernatants were used for Western blotting analysis or immunoprecipitation. Protein concentrations were determined by protein assay kit (Bio-Rad). For the Western blotting analysis, equal amounts of tissue or cell lysates were subjected to SDS**-**PAGE and transferred to PVDF membranes (Millipore, USA). The membrane was then blocked with 5% skimmed milk and probed using an appropriate primary antibody (Table S2) at 4 °C overnight. After the membrane has been washed and it was then incubated with horseradish peroxidase (HRP)**-**conjugated secondary antibodies at room temperature for 1 hr; finally it was developed with Immobilon Western Chemiluminescent HRP Substrate (Millipore).

### Immunofluorescence experiments

Cultured iNs were fixed in 4% paraformaldehyde in phaosphate buffered saline (PBS; 137 mM NaCl, 3 mM KCl, 7 mM Na_2_HPO_4_, 1.5 mM KH_2_PO_4_, pH 7.2–7.4) for 20 min at room temperature, washed three times with PBS, and then incubated in 0.1% Triton X-100 in PBS for 5 min at room temperature. Next the cells were blocked in PBS containing 3% bovine serum albumin (Sigma-Aldrich) and 1% goat serum (Gibco, USA) for 1 hr at room temperature. Each primary antibody was applied at the indicated concentration (Table S2) overnight at 4 °C. Next the cells were washed in PBS three times and then the appropriate secondary antibodies were applied for 1 hr at room temperature. Fluorescent images were captured using a Zeiss microscope and an Andor Zyla cMOS camera and then processed using ImageJ software (NIH, Bethesda, MD, USA).

### RNA isolation, RT-PCR and qPCR analysis

Total RNA was isolated using a Tissue Total RNA Mini Kit (Geneaid, Taipei, Taiwan) by following the manufacturer’s instructions. In-column DNase I digestion was performed to remove genomic DNA contamination. Reverse transcription was then performed using Superscript IV (Invitrogen) and Oligo(dT)_20_ primers by following the manufacturer’s guidelines. For the amplification by polymerase chain reaction (PCR), it was done with a denaturation step at 94 °C for 5 min, followed by 35 cycles of denaturation at 94 °C for 30 sec, primer annealing at 60 °C for 30 sec, and primer extension at 72 °C for 1 min. Upon completion of the cycling steps, a final extension at 72 °C for 5 min was done and then the reaction was stored at 4 °C. The primers for PCR analysis are listed in Table S4. For qPCR analysis, TaqMan PCRs were carried out using FastStart Universal Probe Master Mix (ROX) (Roche) and the StepOnePlus real-time PCR system (Thermo Fisher Scientific). Primers were intron-spanning and designed using the Universal ProbeLibrary Assay Design Center (Roche). The primers and probes for qPCR analysis are listed in Table S5.

### Lactate dehydrogenase (LDH) cytotoxicity assay

Cytotoxicity of HH-F3 was evaluated by measuring LDH activity released in the media after the exposure according to the manufacturer’s instructions (Pierce, USA). Cells without any treatment served as the spontaneously LDH activity control. Following treatment, supernatants from cell without treatment, cells with treatments, and cells treated with lysis buffer were collected and incubated with LDH assay solution accordingly. The optical density values were analyzed at 490 nm by subtracting the reference value at 680 nm using a TECAN sunrise ELISA reader (Tecan Trading AG, Switzerland). LDH positive controls provided in the kit was included in the assay to confirm the success of the assay. The results were expressed as a percentage of treated sample LDH activity (LDH release obtained from treated cells subtracting to spontaneous LDH release obtained from untreated cells) to the total LDH activity (maximal LDH release obtained from lysed cells subtracting to spontaneous LDH release obtained from untreated cells).

### Electrophysiology

Culture iNs were used for electrophysiological analysis on day 28. Whole cell current-clamp recording was performed to characterize intrinsic firing properties by using Multiclamp 700B amplifier (Molecular Devices, Germany). Pipette capacitance was compensated in the cell-attached configuration and pipettes were filled with internal solution containing 120 mM K-gluconate, 24 mM KCl, 0.2 mM EGTA, and 10 mM HEPES (pH was adjusted to 7.3 with KOH). Cells were held at −60 mV and action potential was evoked by injecting currents (10 pA/step). Data were digitized at 10 kHz with a 4 kHz low-pass filter with Digidata 1440 interface (Molecular Devices).

### Intracellular Ca^2+^ measurements

[Ca^2+^]_i_ was measured using fura-2 as previously described^69^. Briefly, cultured iNs were plated on coverglasses. Cells were loaded with 5 µM fura-2/AM (Invitrogen) in the culture medium for 30 min at 37 °C in the dark, then washed twice with the loading buffer containing 150 mM NaCl, 5 mM KCl, 1 mM MgCl_2_, 2.2 mM CaCl_2_, 10 mM HEPES, and 5 mM glucose, pH 7.2. After washing, the coverglass was transferred to an examination chamber. The fast switching excitation wavelengths of 340 nm and 380 nm were provided by a monochromator (Polychrome IV, TILL Photonics GmbH Gräfelfing, Germany), and fluorescence images were acquired through a CCD camera (Micromax YHS1300, Roper Scientific, Trenton, USA) attached to a fluorescence microscope (IX70, Olympus, Japan). The monochromator, CCD camera, and image acquisition were controlled by MetaFluor (Molecular Devices).

### Alkaline phosphatase (AP) staining

The hiPSCs were fixed in 4% paraformaldehyde in PBS for 5 min, and then incubated in 0.1% Triton X-100 in PBS for 5 min at room temperature (RT). After rinse with NTMT substrate buffer (0.1 M Tris-HCl, pH 9.5 containing 0.1 M NaCl, 5 mM MgCl_2_ and 0.1% Triton X-100), the cells were incubated in fresh NTMT buffer containing 0.175 mg/mL of 5-bromo-4-chloro-3-indolyl phosphate (BCIP; Sigma-Aldrich) and 0.45 mg/mL of nitrotetrazolium blue chloride (NBT; Sigma-Aldrich) for 30 min at RT. Samples were then washed two times with PBS and observed under an inverted light microscope.

### *In vitro* embryoid body (EB) formation

hiPSC colonies were treated with collagenase IV (Gibco) and gently scraped off the culture dishes. After centrifugation, cells were resuspended in the EB medium (DMEM/F12 supplemented with 20% KnockOut Serum Replacement (KOSR, Gibco), 2 mM Glutamax, 1% MEM-NEAA and 55 µM 2-mercaptoethanol) and transferred into low attachment dishes. EBs were cultured in a 37 °C incubator with humidified atmosphere of 5% CO_2_. The medium was changed every other day. After 12 days EBs were collected and assessed by RT-PCR for expression of stem cell and differentiation markers.

### Karyotyping

Chromosomal analysis was performed by Giemsa banding (G-banding) analysis at the Cytogenetic Center of Ko’s Obstetrics and Gynecology Clinic, Taipei, Taiwan, following the International System Cytogenetics Nomenclature recommendations.

### T7 endonuclease I (T7E1) assay

Genomic DNA of hiPSCs was extracted using the QuickExtract DNA Extraction Solution (Epicenter) following the manufacturer’s protocol. In brief, the pelleted cells were resuspended in QuickExtract solution and were incubated at 65 °C for 10 min and then 98 °C for 2 min. The sample was stored at –20 °C until use. The genomic region flanking the CRISPR off-target site for each gene was PCR amplified (Table S3), and the products were purified using PCR Cleanup kit (Geneaid) following the manufacturer’s protocol. 400 ng total of the purified PCR products were mixed with 2 μl NEBuffer 2 (NEB) to a final volume of 20 μl and were subjected to a reannealing process to enable heteroduplex formation: 95 °C for 5 min; 95 °C to 85 °C ramping at –2 °C/s; 85 °C to 25 °C at – 0.25 °C/s; and 25 °C hold for 1 min. After reannealing, the products were added with 5 units of T7 Endonuclease I (T7E1) and incubated at 37 °C for 1 hr. The samples were analyzed by DNA gel electrophoresis.

### CellROX deep red assay

Cultured iNs were incubated with 2.5 μM CellROX Deep Red (Invitrogen) for 30 min and 2 μg/mL Hoechst 33342 (Sigma-Adrich) for 15 min. Treatment of 100 μM H_2_O_2_ for 90 min was used to be a positive control. Random images were taken on the Nikon Observer of DIC, CellROX Deep Red and Hoechst 33342 fluorescence.

### Statistics

Data are shown as means ± SEM. Statistical significance was assessed using one-way ANOVA together with the appropriate *post hoc* test; all tests were done using SPSS software (Chicago, USA). Differences where *p* < 0.05 was obtained were considered to be statistically significant.

## Supplementary information

Supplementary Infomation

## Data Availability

All data generated or analyzed during this study are included in this published article and its Supplementary Information.
