# Supplementary material for: Assessing the therapeutic potential of Graptopetalum paraguayense on Alzheimer’s disease using patient iPSC-derived neurons
Source: Sci Rep. 2019 Dec 17;9:19301. doi: 10.1038/s41598-019-55614-9 (PMC6917798; doi:10.1038/s41598-019-55614-9)
Supplement: Supplementary file 1 — Supplementary Infomation [file 41598_2019_55614_MOESM1_ESM.pdf]

Supplementary Information

Assessing the therapeutic potential of *Graptopetalum paraguayense* on Alzheimer's disease using patient iPSC-derived neurons

Authors: Pei-Chun Wu[1], Ming-Ji Fann[1,2], Tu Thanh Tran[1], Shu-Cian Chen[1], Tania Devina[6], Irene Han-Juo Cheng[1,6], Cheng-Chang Lien[1,7], Lung-Sen Kao[1,2], Shuu-Jiun Wang[1,3], Jong-Ling Fuh[1,3], Tsai-Teng Tzeng[4], Chi-Ying Huang[4], Young-Ji Shiao[4,5]*, Yu-Hui Wong[1,*]

Legends of supplementary figures

Supplementary figure 1. Characterization of the AD-iPSC lines. (**a**) Alkaline phosphatase (AP) staining of hiPSC colonies. Scale bar, 100 μm. (**b**) All iPSC lines showed embryonic stem cell-like morphology (Phase) and expressed pluripotent stem cell markers, NANOG (red in upper panels), OCT4 (green in middle panels) and SOX2 (red in lower panels). Scale bar, 50 μm. (**c**) RT-PCR analyses of gene expression in different stages: iPSC and embryoid body (EB) at day 12. The results show expression of stem cell markers, NANOG, OCT4 and SOX2, in iPSCs, and endoderm (AFP and GATA4), mesoderm (T and RUNX1) and ectoderm (NCAM and NESTIN) markers in differentiated EBs. ACTB was used for the internal standard. RT- was used as the negative control. (**d**) Representative G-banding karyotypes of hiPSC lines are shown. At least 20 individual chromosomal spreads were analyzed for each iPSC line at passages 21-44 (iN1: P44; iN2: P39; iAPOE(ε4/ε4): P21; iPS1(P117L): P29; iAPP(D678H): P30; iAPP(corrected): P35). Of 20 metaphases, all were normal with diploid karyotype set of 23 chromosomes.

Supplementary figure 2. Off-target cleavage by CRISPR/Cas9 editing system was not detected in the Crispr/Ca9-edited iAPP(corrected) line. (**a**) Results of T7E1 mismatch detection assay. T7E1 assay was performed at the predicted off-target site with genomic DNA from CRISPR-edited and unedited/parental iPSCs. The predicted results are shown in the right table. (**b**) The accuracy of predicted off-target sites were verified by Sanger sequencing. OT, off target.

Supplementary figure 3. Representative images demonstrating the time-course of the morphological changes when the hiPSCs turn into hiNs over two weeks. Scale bar, 50 μm.

Supplementary figure 4 Characterization of the neuronal properties of induced neurons. (**a**) Representative images of iN cells derived from various iPSC lines 4 weeks after induction. iN cells were detected using antibodies against MAP2, Smi312, NeuN and AnkG as indicated. Scale bar, 50 μm. (**b, c**) The bar graphs present average axonal length at D9 (B, from six batches) and dendritic length at D28 (C, from two batches). Each dot presents the axonal or dendritic length of one neuron. The number of cells analyzed is showed in parentheses.

Supplementary figure 5. Characterization of the properties of induced neurons by real-time RT-PCR. (**a**) Heatmap of RT-qPCR analysis of selected genes using the iPSCs and iPSC-derived neurons at D28. Levels are normalized against RPL13A mRNA levels as an internal control. (**b**) Quantitative results of the mRNA levels of the AD-related genes in the iPSCs and iPSC-derived neurons at D28. The levels are normalized against RPL13A mRNA levels as an internal control (n = 4 and 6 independent experiments for iPSC and iN_D28, respectively).

Supplementary figure 6. Representative images of living iNs at D28 (five days after HH-F3 treatment). No obvious cell death or abnormal cell morphology was found by phase-contrast microscope after HH-F3 treatment. Scale bar, 50 μm.

Supplementary figure 7. The effects of GP extract on the secretion of Aβ in iNs from multiple AD patients carrying different mutations. (**a**) iN1, iAPP(D678H) and iAPP(corrected) derived neurons were exposed to 5, 10, 20 and 50 μg/mL of HH-F3 for 5 days and the dose effect of HH-F3 on the Aβ1-40 and Aβ1-42 concentrations using ELISA assays was measured and presented as pg/μg protein. The results were obtained from four independent experiments. (**b**) The effect of HH-F3 on iN2, iPS1(P117L) and iAPOE(ε4/ε4)-derived neurons were also elucidated. Cells were treated with 50 μg/mL HH-F3 and the amount of extracellular Aβ1-40 and Aβ1-42, presented as pg/μg protein, was measured. The results were obtained from five to six independent experiments. The bar graphs present average secretion of Aβ. Each dot presents individual secretion after treatments, and the dots coded with the same colors in each graph indicate that they were obtained from same differentiation.

Supplementary figure 8. The effects of HH-F3 on the p-Tau at T181 and S262. (**a-c**) Western blotting analysis was used to monitor the expression of p-Tau at Thr181 and Ser262, as well as total Tau in iN1, iAPP(D678H) and iAPP(corrected)-derived neurons after treatment with 5, 10, 20 and 50 μg/mL of HH-F3 and 50 ng/mL of compound E for 5 days. (**d-f**) Quantitative results of (**a-c**) are shown as means ± SEM; n = 3-4 independent batches of differentiation. The intensity of the p-Tau signals was normalized against total Tau (**d, e**), and total Tau signals was normalized against GAPDH (**f**). The obtained values were normalized against the control. No statistically significant changes were observed, except for an increase in the level of p-Tau phosphorylated at Thr181 when 50 μg/mL of HH-F3 was used. * $p < 0.05$ by one-way ANOVA with Fisher's least significant difference method. (**g-k**) Panels **g-k** show representative Western blotting from the control and AD-iPSC derived neurons treated with 50 μg/mL of HH-F3 and 50 ng/mL of compound E (CPD-E) for 5 days. (**l-n**) Quantitative analysis showed a significant reduction in p-Tau phosphorylated at Thr181 in iPS1(P117L), but elevation of p-Tau phosphorylated at Ser262 in iAPOE(ε4/ε4). Total Tau did not show significant alterations. The results are shown as means ± SEM; n = 3-4 independent batches of differentiation. * $p < 0.05$ by one-way ANOVA with Fisher's least significant difference method.

Supplementary figure 9. The effects of memantine on extracellular Aβ levels and Tau protein phosphorylation. The iPSCs were induced to differentiate into neural cells by Ngn2 expression. 23 days after differentiation, the cells were treated with 50 μg/mL HH-F3 extract, 10 μM memantine, or vehicle, all for 5 days. The media were then analyzed by ELISA to measure level of Aβ (**a**) and lysates were harvested for Western blot using antibody against p-Tau at position Ser396 (**b**). Quantitative results are shown as means (n = 2 independent batches of differentiation).

Supplementary figure 10. The effects of GP extract on the intracellular ROS level. iN2 derived neurons were treated with 50 ng/mL CPD-E and 50 μg/mL HH-F3 for 5 days, and 100 μM H_2O_2 for 90 min as a positive control. Intracellular ROS was observed with CellROX Deep Red. Representative images of DIC and CellROX fluorescence were shown. Scale bar: 20 μm.

Figure S1

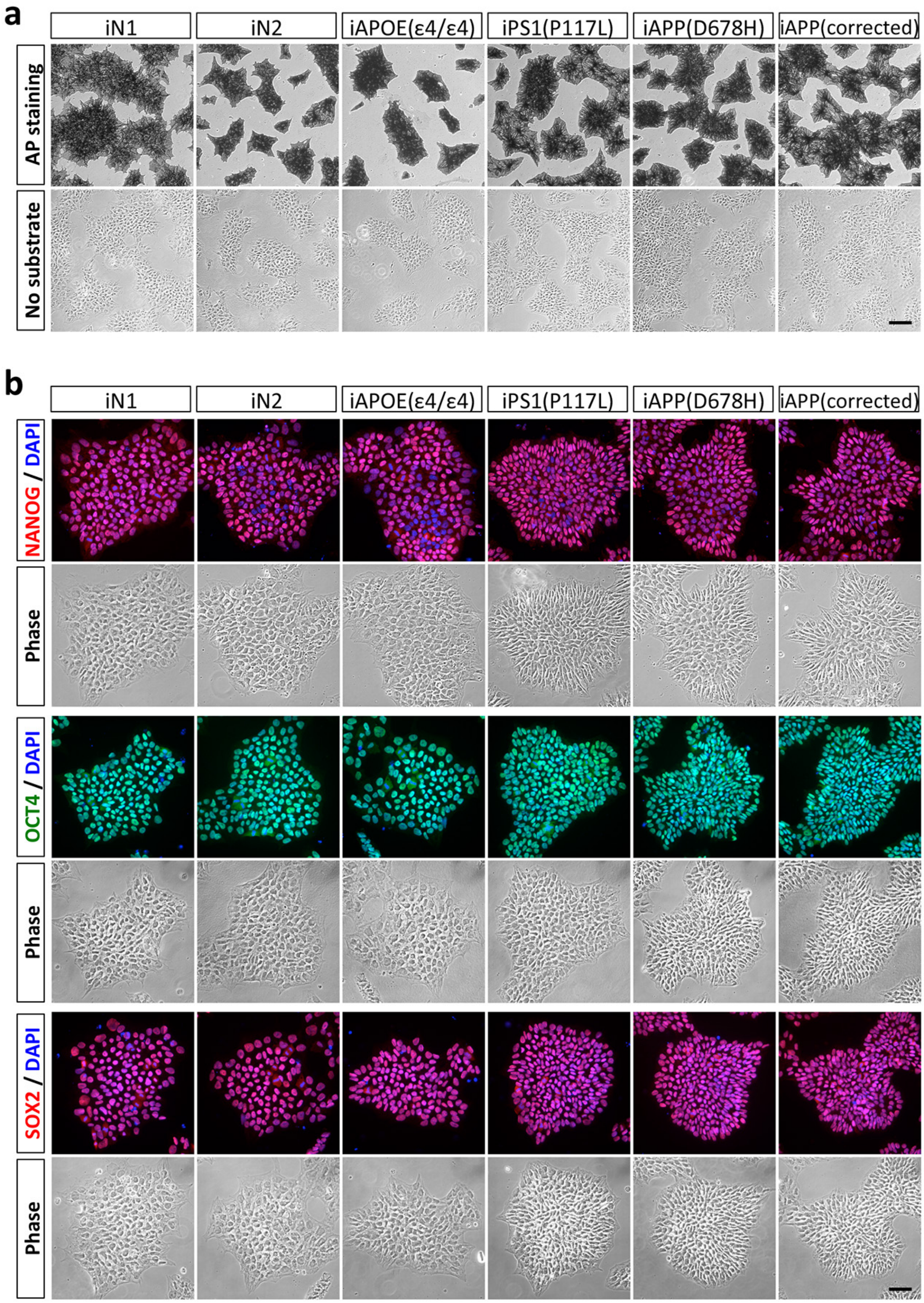
a
iN1
iN2
iAPOE(ε4/ε4)
iPS1(P117L)
iAPP(D678H)
iAPP(corrected)
AP staining
No substrate
b
iN1
iN2
iAPOE(ε4/ε4)
iPS1(P117L)
iAPP(D678H)
iAPP(corrected)
NANOG / DAPI
Phase
OCT4 / DAPI
Phase
SOX2 / DAPI
Phase

Figure S1 (continued)

c

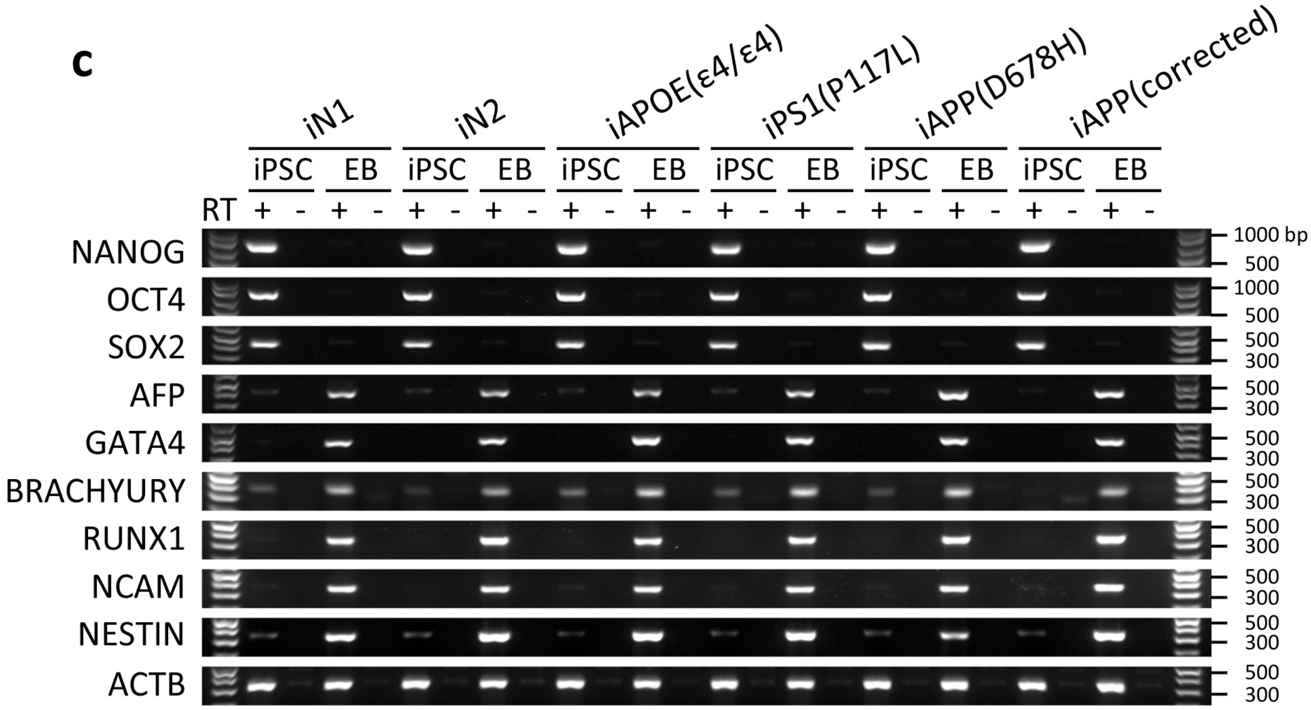

d

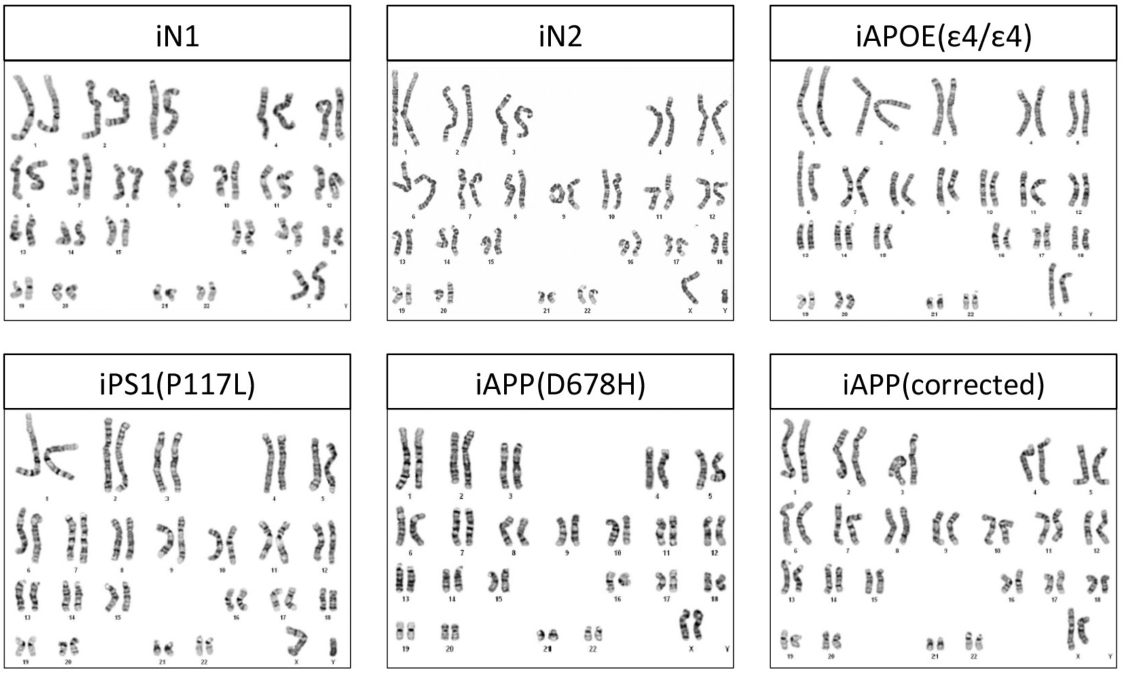

Figure S2

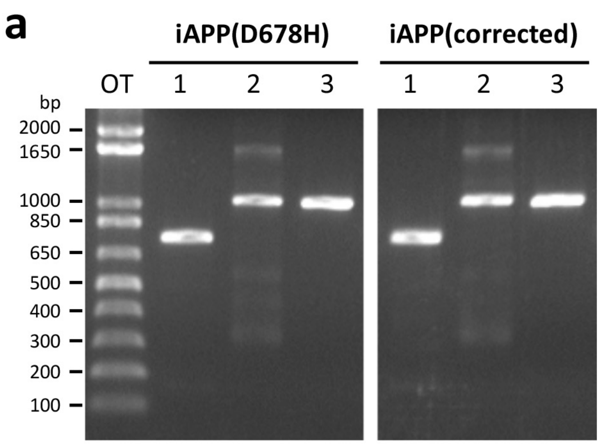

	WT	INDEL
**OT1**	726 (bp)	390, 336
**OT2**	987	584, 403
**OT3**	914	587, 327

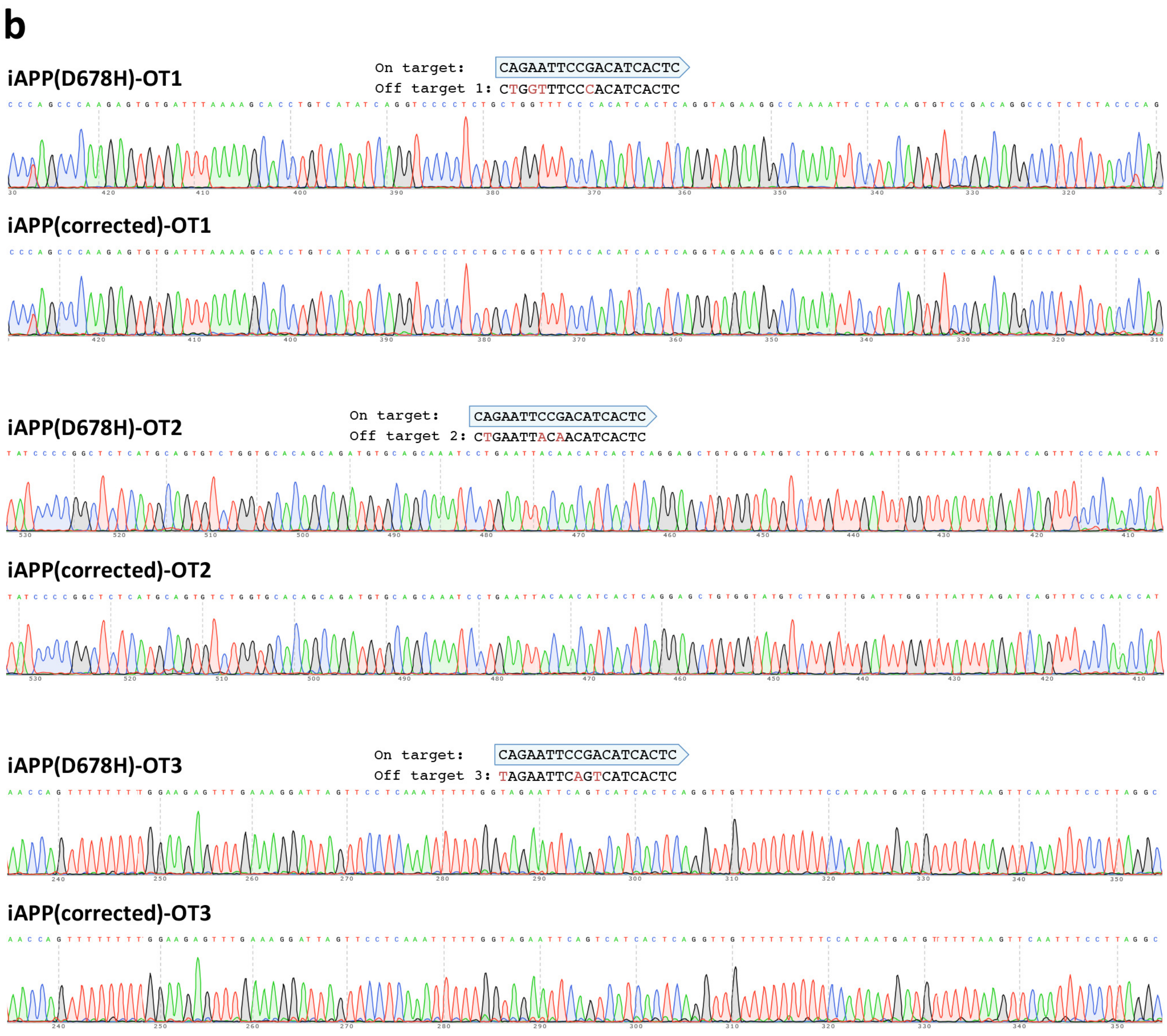

Figure S3

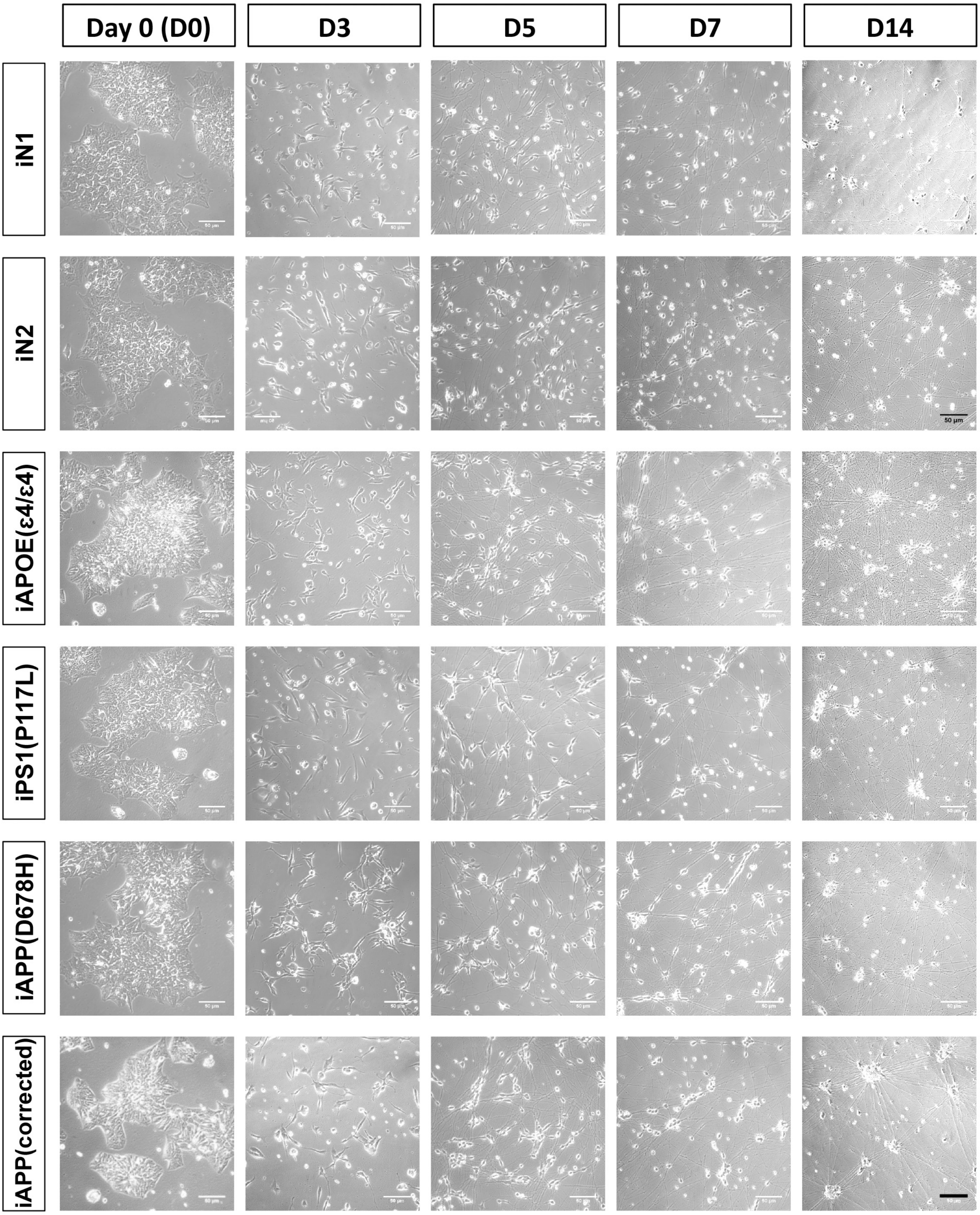

Day 0 (D0)
D3
D5
D7
D14
iN1
iN2
iAPOE(ε4/ε4)
iPS1(P117L)
iAPP(D678H)
iAPP(corrected)
50 μm

Figure S4

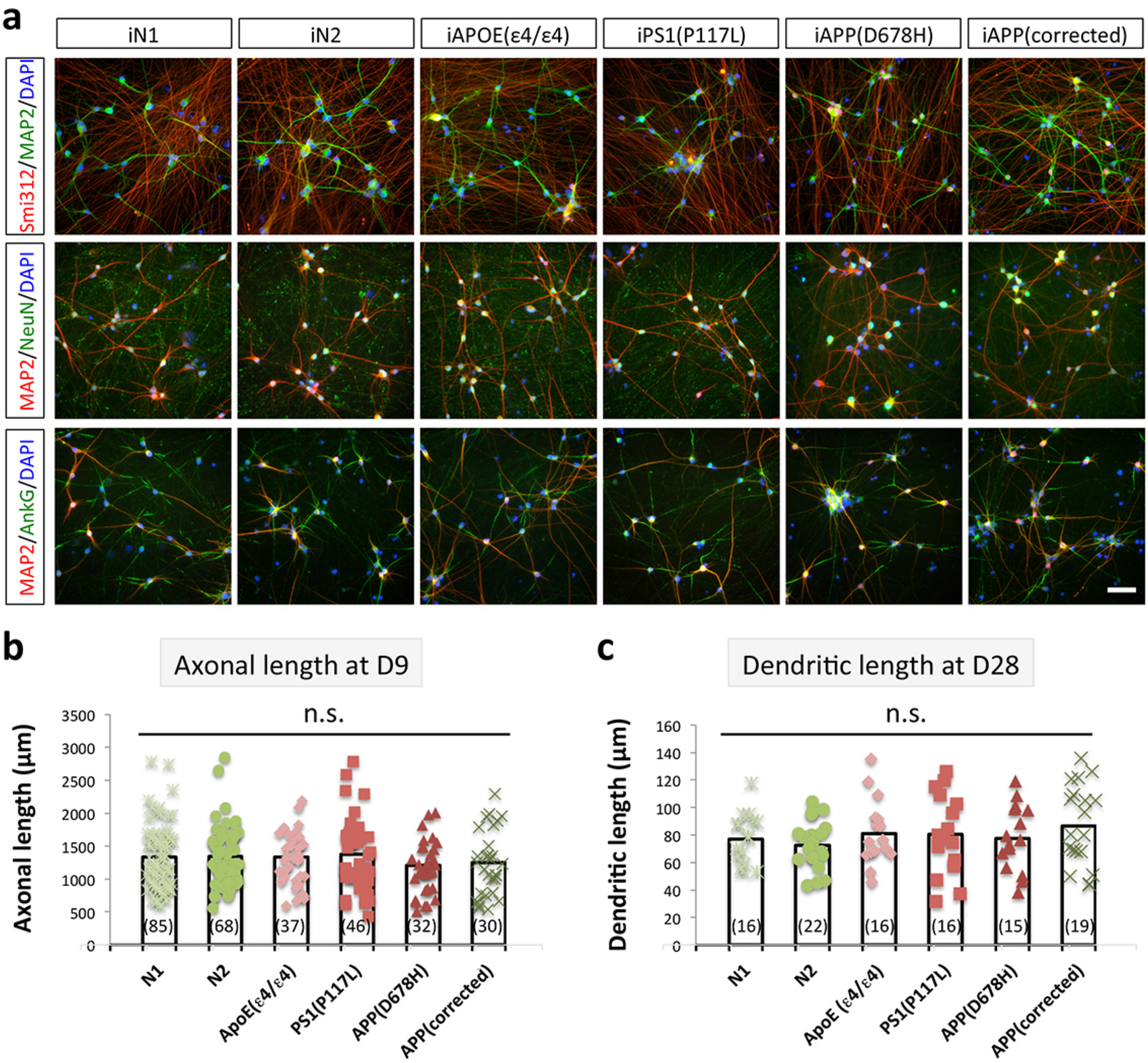
a
iN1
iN2
iAPOE(ε4/ε4)
iPS1(P117L)
iAPP(D678H)
iAPP(corrected)
Smi312/MAP2/DAPI
MAP2/NeuN/DAPI
MAP2/AnkG/DAPI
b
Axonal length at D9
n.s.
Axonal length (μm)
3500
3000
2500
2000
1500
1000
500
0
(85)
(68)
(37)
(46)
(32)
(30)
N1
N2
ApoE(ε4/ε4)
PS1(P117L)
APP(D678H)
APP(corrected)
c
Dendritic length at D28
n.s.
Dendritic length (μm)
160
140
120
100
80
60
40
20
0
(16)
(22)
(16)
(16)
(15)
(19)
N1
N2
ApoE (ε4/ε4)
PS1(P117L)
APP(D678H)
APP(corrected)

Figure S5

a

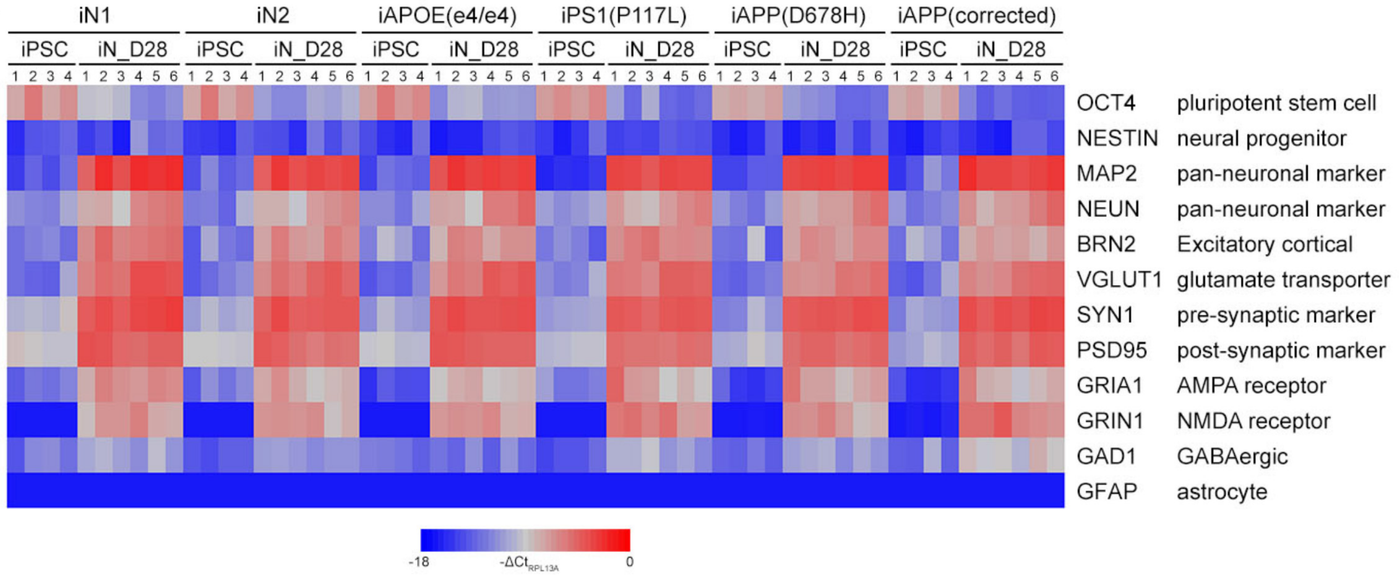
iN1
iN2
iAPOE(e4/e4)
iPS1(P117L)
iAPP(D678H)
iAPP(corrected)
iPSC
iN_D28
OCT4 pluripotent stem cell
NESTIN neural progenitor
MAP2 pan-neuronal marker
NEUN pan-neuronal marker
BRN2 Excitatory cortical
VGLUT1 glutamate transporter
SYN1 pre-synaptic marker
PSD95 post-synaptic marker
GRIA1 AMPA receptor
GRIN1 NMDA receptor
GAD1 GABAergic
GFAP astrocyte
-18
-ΔCt RPL13A
0

b

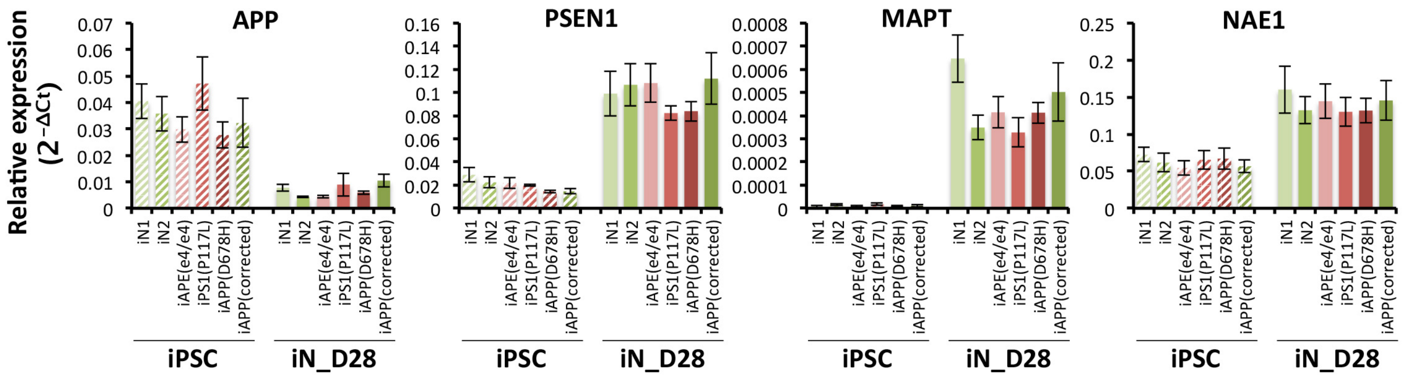
Relative expression (2-ΔCt)
APP
PSEN1
MAPT
NAE1
iN1
iN2
iAPE(e4/e4)
iPS1(P117L)
iAPP(D678H)
iAPP(corrected)
iPSC
iN_D28

Figure S6

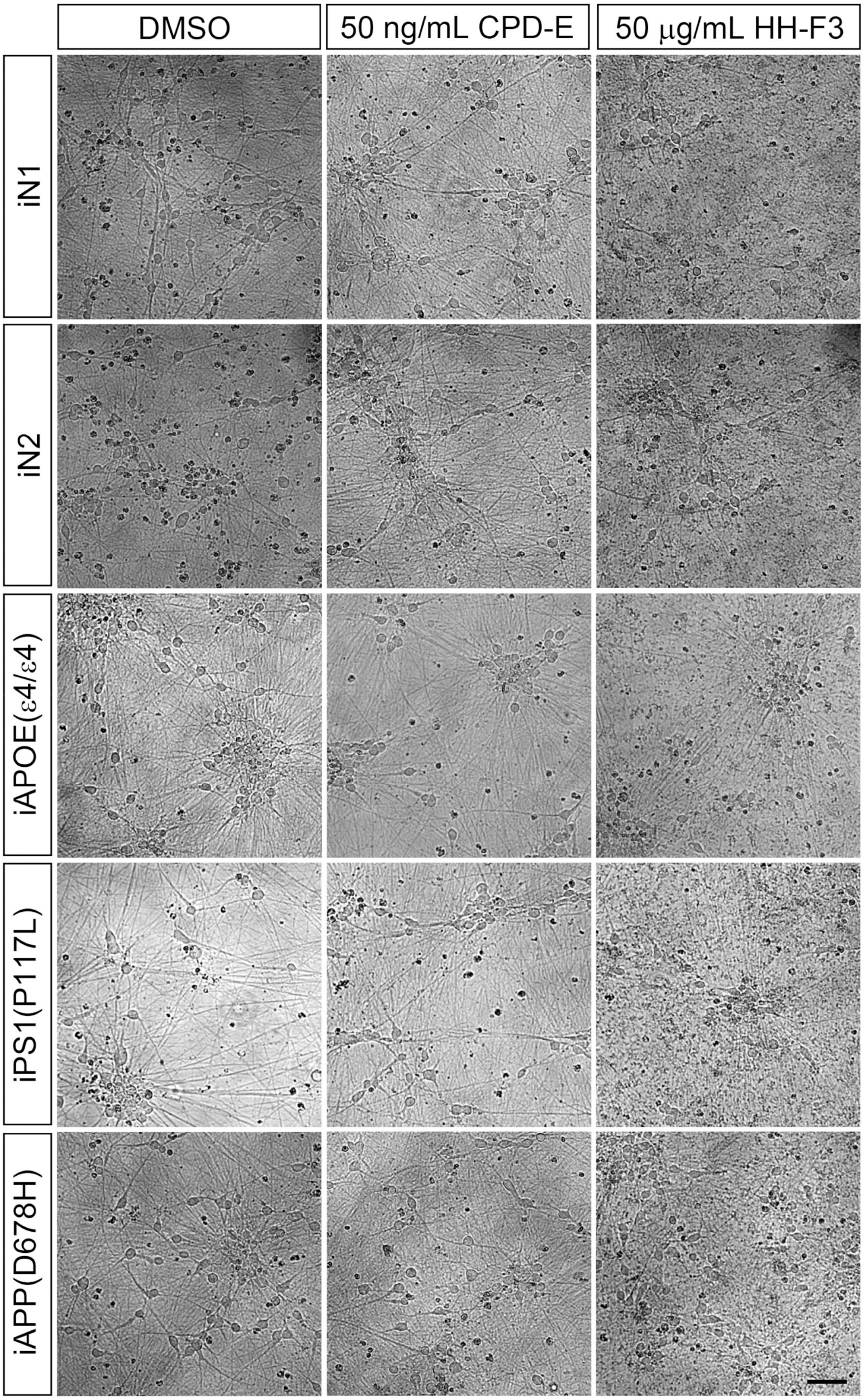
DMSO
50 ng/mL CPD-E
50 μg/mL HH-F3
iN1
iN2
iAPOE(ε4/ε4)
iPS1(P117L)
iAPP(D678H)

Figure S7

a (related to Fig. 3b)

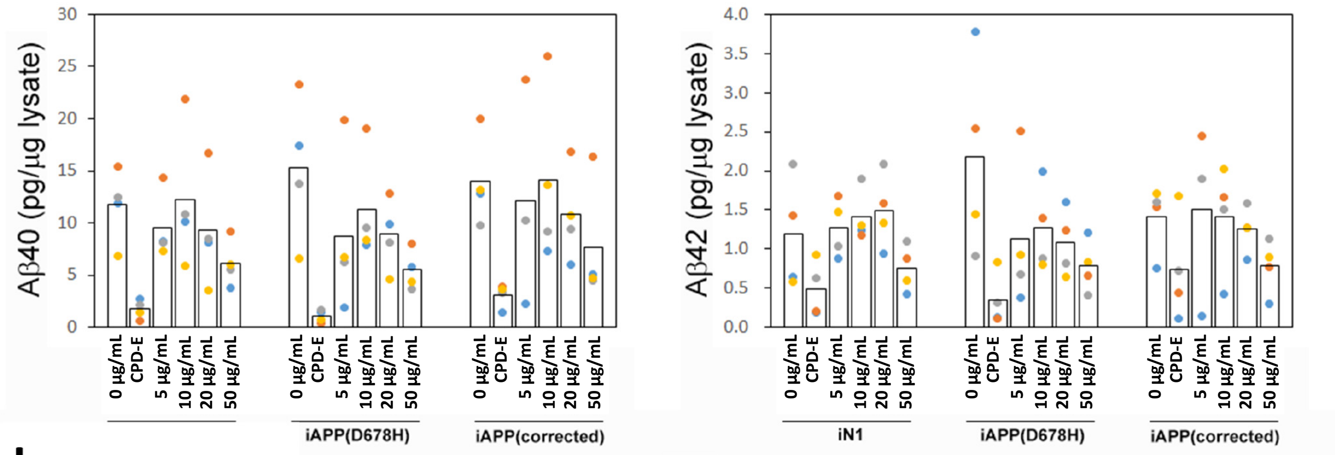

b (related to Fig. 4b)

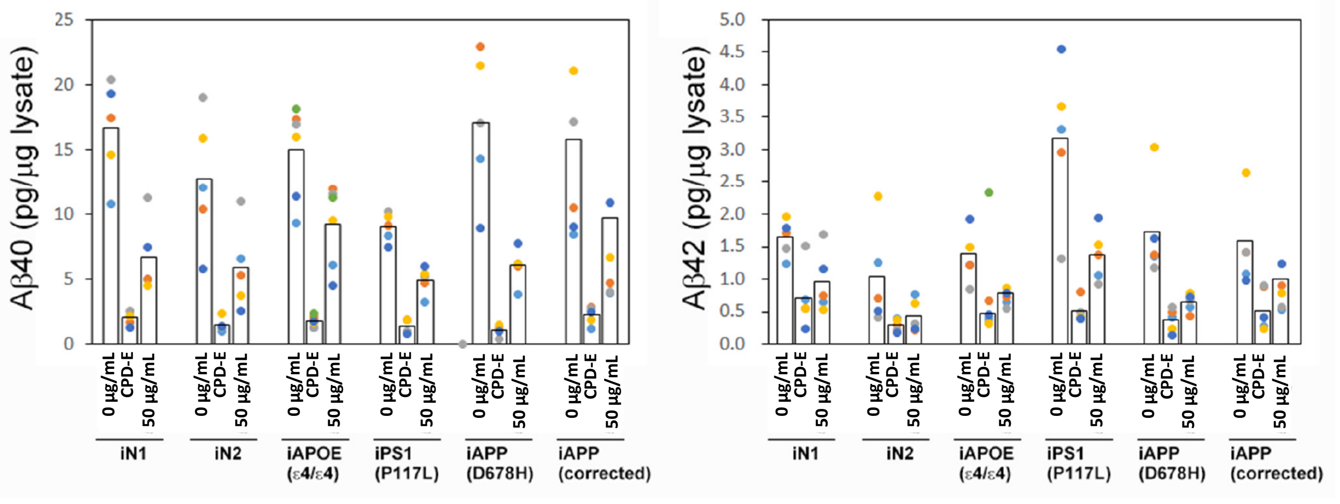

Figure S8

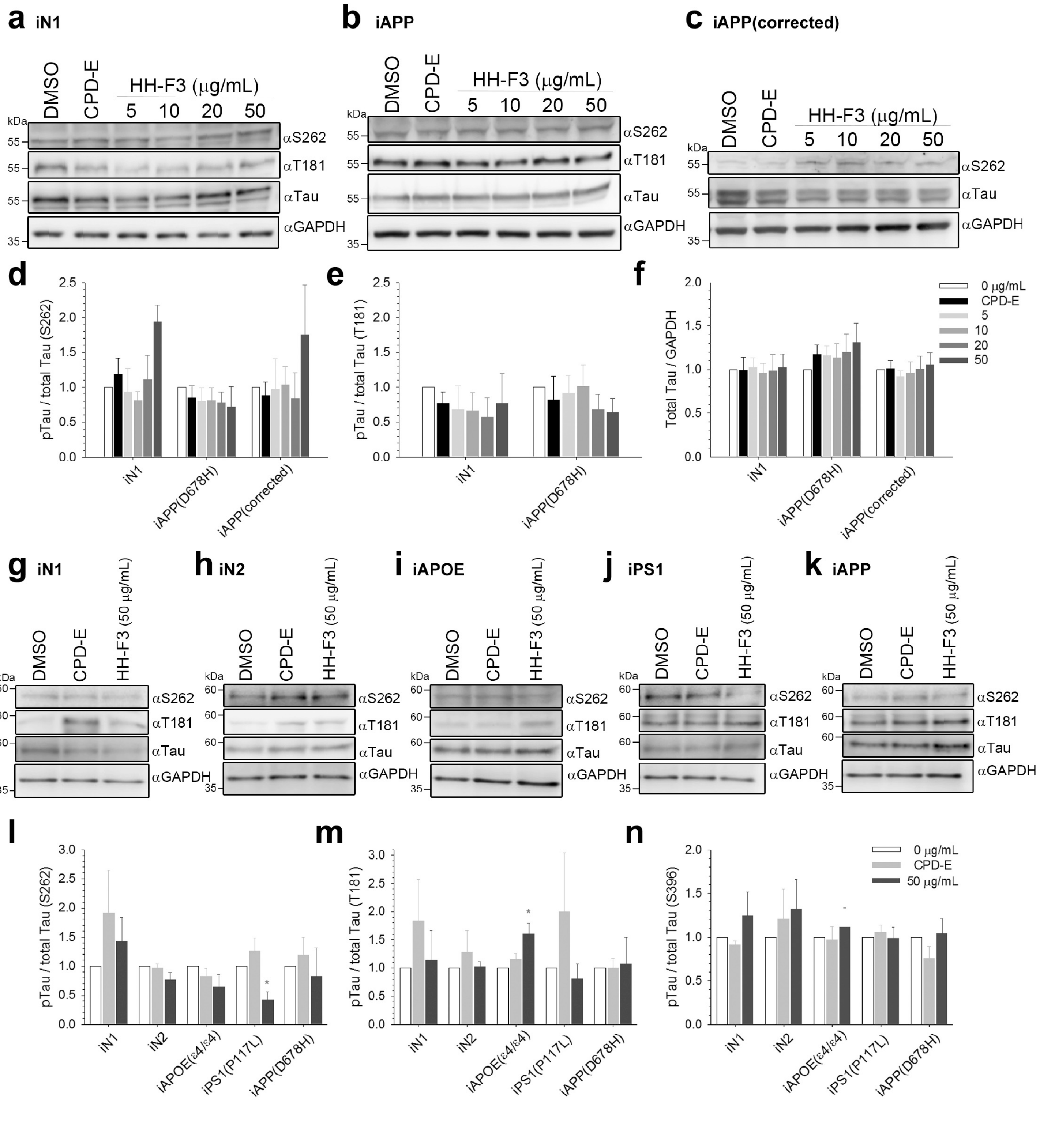
a iN1
b iAPP
c iAPP(corrected)
DMSO
CPD-E
HH-F3 (μg/mL)
5
10
20
50
αS262
αT181
αTau
αGAPDH
d
e
f
pTau / total Tau (S262)
pTau / total Tau (T181)
Total Tau / GAPDH
0 μg/mL
CPD-E
iN1
iAPP(D678H)
iAPP(corrected)
g iN1
h iN2
i iAPOE
j iPS1
k iAPP
HH-F3 (50 μg/mL)
l
m
n
pTau / total Tau (S396)
50 μg/mL
iN2
iAPOE(ε4/ε4)
iPS1(P117L)

Figure S9

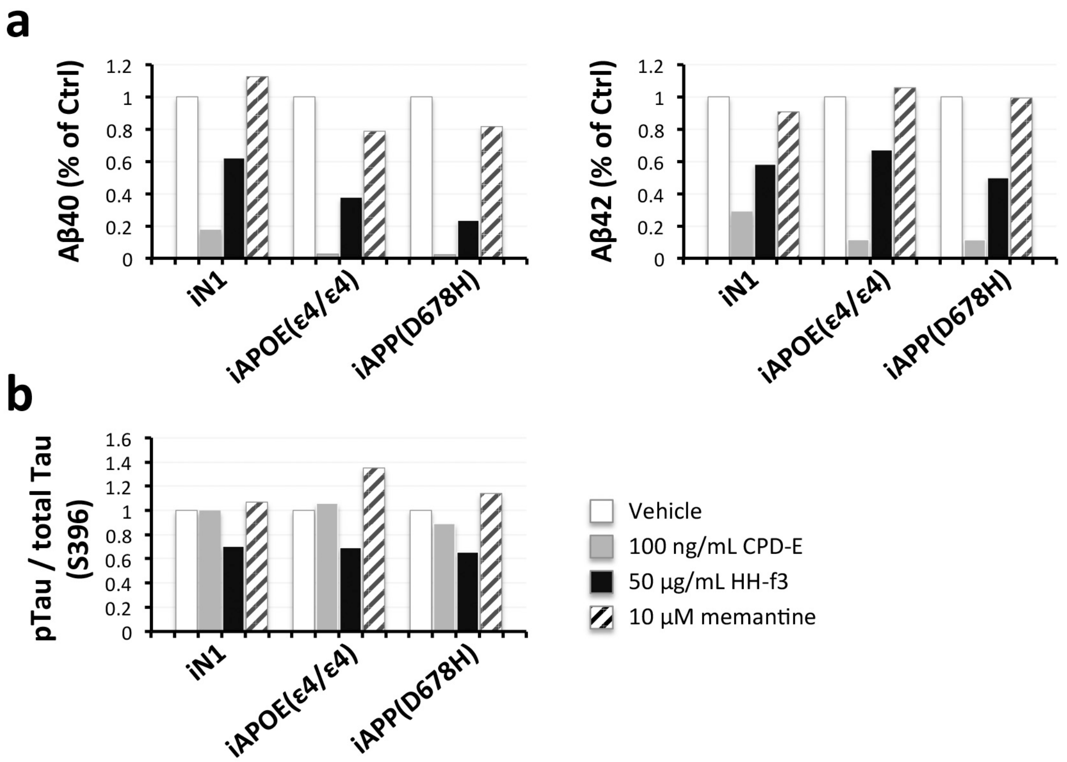

Figure S10

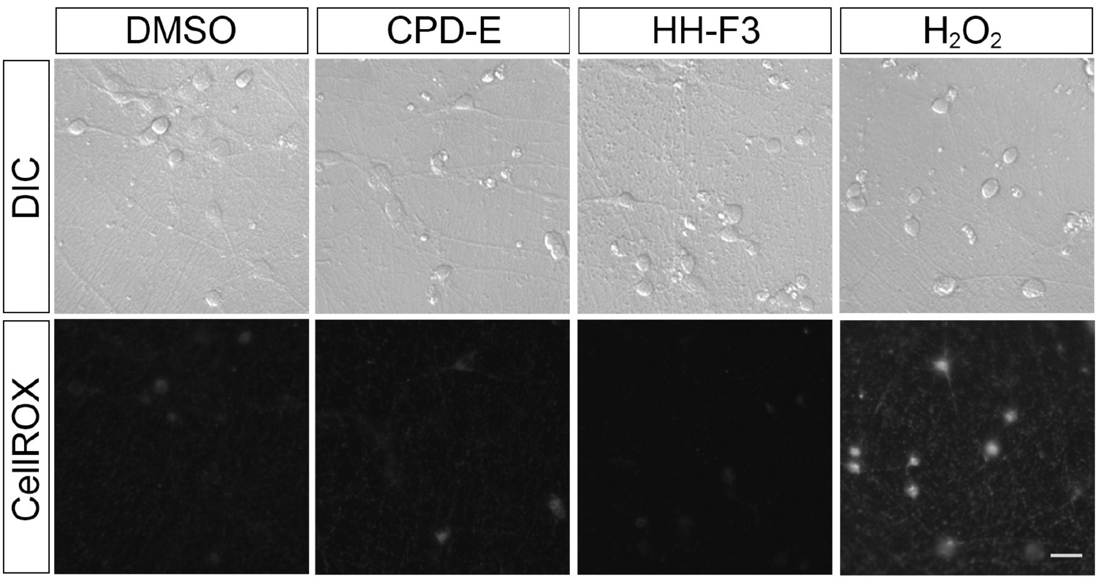

Table S1. Publications used for AD therapeutic prediction by gene set enrichment analysis (GSEA)

Author	Journal	Brain regions	Array
Wu, Z. et al.	*Nat Med* 11, 959-965 (2005) [41]	Brain endothelial cells	Affymetrix HG-U95A
Blalock, E. M. et al.	*Proc Natl Acad Sci U S A* 101, 2173-2178 (2004) [34]	Hippocampus	Affymetrix HG-U133A
Lu, T. et al.	*Nature* 429(6994):883-91 [38]	Frontal cortex	Affymetrix HG-U95AV2
Hata, R. et al.	*Biochem Biophys Res Commun* 284, 310-316 (2001) [37]	Hippocampus/ parietal cortex	Human UniGem V microarray
			cDNA chips (Incyte Pharma-ceuticals)
Ricciarelli, R. et al.	*IUBMB Life* 56, 349-354 (2004) [39]	Middle frontal gyrus (Gfm)	Atlas Human 12K Microarray (array lot# 2070569)
Bossers, K. et al.	*Brain* 133, 3699-3723 (2010) [35]	Prefrontal cortex	Agilent 44K Whole Human Genome arrays
Ginsberg, S. D. et al.	*Biol Psychiatry* 68, 885-893 (2010) [36]	Hippocampal CA1	Custom-DesignedcDNA / ArrayPlatforms
Williams, C. et al.	*PLoS One* 4, e4936 (2009) [40]	prefrontal cortex	Affymetrix HG-U133A

Table S2. Antibodies used in this study

Antibodies (immunofluorescent staining)	Host	Vendor	Cat. No.	Dilution
AnkG	Rabbit	Invitrogen	PA5-83418	1:400
MAP2	Rabbit	Millipore	AB5622	1:2000
MAP2	Mouse	Millipore	MAB378	1:1000
Nanog	Rabbit	CST	3580	1:200
NeuN	Mouse	Abcam	ab104224	1:500
OCT4	Rabbit	GeneTex	GTX101497	1:1000
TUJ1	Mouse	BioLegend	801201	1:2000
SMI312	Mouse	BioLegend	837904	1:1000
SOX2	Rabbit	Millipore	AB5603	1:500
VGluT2	Rabbit	Frontier Institute	VGluT2-Rb-Af670	1:200

Antibodies (secondary)	Host	Vendor	Cat. No.	Dilution
Goat anti-Mouse IgG (H+L) Highly Cross-Adsorbed Secondary Antibody, Alexa Fluor Plus 488	Goat	Invitrogen	A32723	1:500
Goat anti-Mouse IgG (H+L) Highly Cross-Adsorbed Secondary Antibody, Alexa Fluor Plus 555	Goat	Invitrogen	A32727	1:500
Goat anti-Rabbit IgG (H+L) Highly Cross-Adsorbed Secondary Antibody, Alexa Fluor Plus 488	Goat	Invitrogen	A32731	1:500
Goat anti-Rabbit IgG (H+L) Highly Cross-Adsorbed Secondary Antibody, Alexa Fluor Plus 555	Goat	Invitrogen	A32732	1:500

Antibodies (western)	Host	Vendor	Cat. No.	Dilution
GAPDH	Mouse	OriGene Technologies	ACR001PS	1:10000
MAP2	Rabbit	Millipore	AB5622	1:2000
OCT4	Rabbit	GeneTex	GTX101497	1:2000
Phospho-Tau (Thr181) (D9F4G)	Rabbit	Cell Signaling Technology	12885	1:1000
Phospho-Tau (Ser214)	Rabbit	Millipore	AB9472	1:1000
Phospho-Tau (Ser262)	Rabbit	Santa Cruz	sc-32828	1:1000
Phospho-Tau (Ser396) (PHF13)	Mouse	Cell Signaling	9632	1:1000
Synaptophysin	Mouse	Millipore	MAB5258	1:1000
Tau Antibody (H-150)	Rabbit	Santa Cruz	sc-5587	1:2000

Antibodies (secondary)	Host	Vendor	Cat. No.	Dilution
Mouse IgG-heavy and light chain antibody	Goat	Bethyl Laboratories	A90-116P	1:10000
Rabbit IgG-heavy and light chain antibody	Goat	Bethyl Laboratories	A120-101P	1:10000

Table S3. Primers for PCR of predicted off-target fragments

Fragment	Primer name	Sequence (5' to 3')	Predicted size (bp)
OT1	APPgRNA#1_offT1_f	GATGGGAGTGTCTGCCTTC	726
	APPgRNA#1_offT1_r	AGACCGTGTCTGGCTCTTG	
OT2	APPgRNA#1_offT2_f	CATGGCGAAATCCTGTCTC	987
	APPgRNA#1_offT2_r	TATTGGAAAGGAGCCCAGC	
OT3	APPgRNA#1_offT3_f2	CCTGGGAAACATATTGAGACC	914
	APPgRNA#1_offT3_r2	GTTAACAATCCACCAATCAGC	

Table S4. Primers for RT-PCR

Gene	Primer name	Sequence (5' to 3')	Predicted size (bp)
NANOG	hNanog-for	AATACCTCAGCCTCCAGCAG	747
	hNanog-rev1	AACCTCGCTGATTAGGCTCC	
OCT3/4	hOct4-for	GCTGGAGAAGGAGAAGCTGG	795
	hOct4-rev1	AAACCCTGGCACAAACTCCA	
SOX2	hSox2-for1	GAGAAAACCTGGGGAGGGTG	434
	hSox2-rev1	CACAGAGATGGTTCGCCAGT	
AFP	hAFP-for	GAAAAATGGCAGCCACAGCA	395
	hAFP-rev	GCAGCATTTCTCCAACAGGC	
GATA4	hGATA4-for	CTGAAGCTCTCCCCACAAGG	409
	hGATA4-rev	GGGGACAAGGACATCTTGGG	
BRACHYURY	hT-for	GTCTATGTGGACCCACGCAA	371
	hT-rev	CCACCTGCATCCCTCAGAAG	
RUNX1	hRunx1-for	CCTGGGATCCATTGCCTCTC	365
	hRunx1-rev	CGCCGTAGTACAGGTGGTAG	
NCAM	hNCAM-for	TGTCCCCTGCAACTACAACC	347
	hNCAM-rev	AGTCCTCCGTTGGGTGTTTC	
NESTIN	hNestin-for	GCGTTGGAACAGAGGTTGG	333
	hNestin-rev	CAGGACTGGGAGCAAAGATCC	
ACTB	hACTB-for	TGGCACCCAGCACAATGAAG	337
	hACTB-rev	CTGTCACCTTCACCGTTCCA	

Table S5. QPCR primers and TaqMan® probes

Marker	Gene	Assesion #	Probe #	Sequence (5' to 3')
reference(BMC)	**RPL13A**	NM_012423.2	28	(F) CAAGCGGATGAACACCAAC
				(R) TGTGGGGCAGCATACCTC
stem cell	**POU5F1(Oct4)**	NM_002701.4	60	(F) CTTCGCAAGCCCTCATTTC
				(R) GAGAAGGCGAAATCCGAAG
NSC	**Nestin**	NM_006617.1	1	(F) TGCGGGCTACTGAAAAGTTC
				(R) GCTCTGTAGGCCCTGTTTCTC
general neuron	**Map2**	NM_002374.3	62	(F) CCTGTGTTAAGCGGAAAACC
				(R) AGAGACTTTGTCCTTTGCCTGT
general neuron	**RBFOX3(NeuN)**	NM_001082575.1	66	(F) CCCTCCGACCCTACAGAGA
				(R) CCACGTCTAAAATTTTTCCGAAT
excitatory cortical neuron	**POU3F2(BRN2)**	NM_005604.2	47	(F) AATAAGGCAAAAGGAAAGCAACT
				(R) CAAAACACATCATTACACCTGCT
AMPA receptor	**GRIA1**	NM_000827.3	62	(F) CAAGGGATCGACATCCAGAG
				(R) CTGCACGTTTCCTGTTAAACC
AMPA receptor	**GRIA2**	NM_000826.3	1	(F) AGAAAGATGGTCAACACTGGAAG
				(R) CATCACTTGAACGGCATCAT
NMDA receptor	**GRIN1**	NM_000823.6	67	(F) GCTCAGAAACGCCTGGAG
				(R) TGGGTCAAACTGCAGCAC
NMDA receptor	**GRIN2A**	NM_001134408.2	42	(F) TCTATGATCATGGCTGACAAGG
				(R) CTGCATGATCTTCAGCATGAC
GABAergic neuron	**GAD1, transcript**	NM_013445.3	1	(F) CAACTTGGAGCTCTCTGACCA
				(R) TGAAAAATCGAGGATGACCTG
glutamate transporter	**VGLUT1**	NM_020309.3	29	(F) AGAGCGCGAAACTCATGAAC
				(R) GCAGCAGGTAGAACGTCCAG
pre-synaptic marker	**SYN1**	NM_006950.3	67	(F) GGACGTCAGTGTCAGGGAAC
				(R) GTGTCCACCCACAGCTTGTA
post-synaptic marker	**PSD95**	U83192.1	50	(F) CGCTACCAAGATGAAGACACG
				(R) GGGAGAATTGGCCTGGTT
astrocyte	**GFAP**	NM_002055.4	29	(F) AGAGGGACAATCTGGCACA
				(R) CAGCCTCAGGTTGGTTTCAT
AD	**APP**	NM_000484.3	66	(F) TTGCCCGAGATCCTGTTAAA
				(R) TACTTGTCAACGGCATCAGG
AD	**Psen1**	NM_000021.3	29	(F) CCTCAACAATGGTGTGGTTG
				(R) TTGTGACTCCCTTTCTGTGCT
AD	**Mapt**	NM_016835.4	6	(F) GGACTGGAAGCGATGACAA
				(R) GGCTAAGGCAAGGCCTATTT
AD	**Nae1**	NM_003905.3	50	(F) CTTCAGAGATTTGATTAGACAAGGAA
				(R) TCTTCAATACTGCTTGGGATCTG
